# The earliest Tyrannida (Aves, Passeriformes), from the Oligocene of France

**DOI:** 10.1038/s41598-020-66149-9

**Published:** 2020-06-17

**Authors:** Ségolène Riamon, Nicolas Tourment, Antoine Louchart

**Affiliations:** 1 Univ Lyon, Univ Lyon 1, ENSL, CNRS, LGL-TPE, F-69622 Villeurbanne, France; 2 13012, Marseille, France

**Keywords:** Evolution, Zoology, Ecology

## Abstract

Passeriformes is the most diverse bird order. Nevertheless, passerines have a remarkably poor early fossil record. In addition, high osteological homoplasy across passerines makes partial specimens difficult to systematically assign precisely. Here we describe one of the few earliest fossil passerines, from the early Oligocene (ca 30 Ma) of southern France, and one of the best preserved and most complete. This fossil can be conservatively assigned to Tyrannida, a subclade of the New World Tyranni (Suboscines), i.e. of the Tyrannides. A most probably stem-representative of Tyrannida, the new fossil bears strong resemblance with some manakins (Pipridae), possibly due to plesiomorphy. Furthermore, it yields a new point of calibration for molecular phylogenies, already consistent with the age of the fossil. Tyrannida, and the more inclusive Tyrannides, are today confined to the New World. Therefore, the new fossil calls for scenarios of transatlantic crossing during or near the Oligocene. Later, the European part of the distribution of the Tyrannida disappeared, leading to a relictual modern New World distribution of this clade, a pattern known in other avian clades. The history of Tyrannida somehow mirrors that of the enigmatic *Sapayoa aenigma*, sole New World representative of the Eurylaimides (Old World Tyranni), with transatlantic crossing probably caused by similar events.

## Introduction

The order Passeriformes (Aves) comprises 59% of the extant bird diversity, i.e. 6,493 over ca. 11,000 species^1^. They comprise the basal Acantisittidae (two species), sister to the Eupasseres which in turn comprises the Tyranni (previously called Suboscines; 1,407 species), and the Passeri (previously called Oscines; 5,084 species). Molecular studies show that, as the sister clade to Psittaciformes (parrots and allies), Passeriformes originated in the earlier part of the Paleogene, and most of the extant families diverged near the Eocene-Oligocene-limit, i.e. some of them should be approximately as old as 30 million years (Ma)^2–4^. Nevertheless, the fossil record of passerine birds remains exceedingly poor prior to the middle Miocene, and increasingly toward earlier times. This taphonomic bias explains the difficulty in finding early specimens representing extant passerine clades. Among the few pre-Miocene published specimens, most are fragmentary^5–11^, and even the three more complete specimens, on slab, are rather poorly preserved and prove difficult to identify with some precision. The latter specimens are all from the European early Oligocene: *Wieslochia weissi* (Germany^12,13^), *Jamna szybiaki* and *Resoviaornis jamrozi* (Poland^14,15^). This difficulty is also explained by (i) high apparent homoplasy observed on osteological characters within the Passeriformes^12,13^, and (ii) the difficulty to compare fossils with a sufficiently large, representative sample of extant taxa, passerine clades being so rich at specific and generic levels. Some early fossils have been referred to Tyranni indet., others to Passeri indet., and the remaining to either the preceding taxa or possibly stem Eupasseres or stem passerines^5–15^. All these fossils date to the Oligocene of France, Germany and Poland in Europe. In addition, late Oligocene fossils of logrunner (Oscines, Orthonychidae) are known from Australia^16^. As for the older, possible passerine remains from the lower Eocene of Australia^17,18^, they are fragmentary (one proximal carpometacarpus and one distal tibiotarsus) and considered to be either not sufficiently diagnostic of the Passeriformes^12,19^ or possibly Passeriformes outside Eupasseres^20^. Here we describe one of the earliest fossils on slab of a passerine bird, nearly complete, from the early Oligocene of the Luberon (Alpes-de-Haute-Provence, France). Its exceptional state of preservation allows for its identification as the oldest Passeriformes assignable to a modern subgroup of the Tyrannides (the latter being sometimes called “New World Tyranni”). This fossil provides the earliest calibration point for a subclade of the Tyranni. In addition, it yields evidence of an American passerine element in this locality, calling for several plausible paleobiogeographical scenarios.

## Results

### Assignment to the Passeriformes

**Figure 1:**
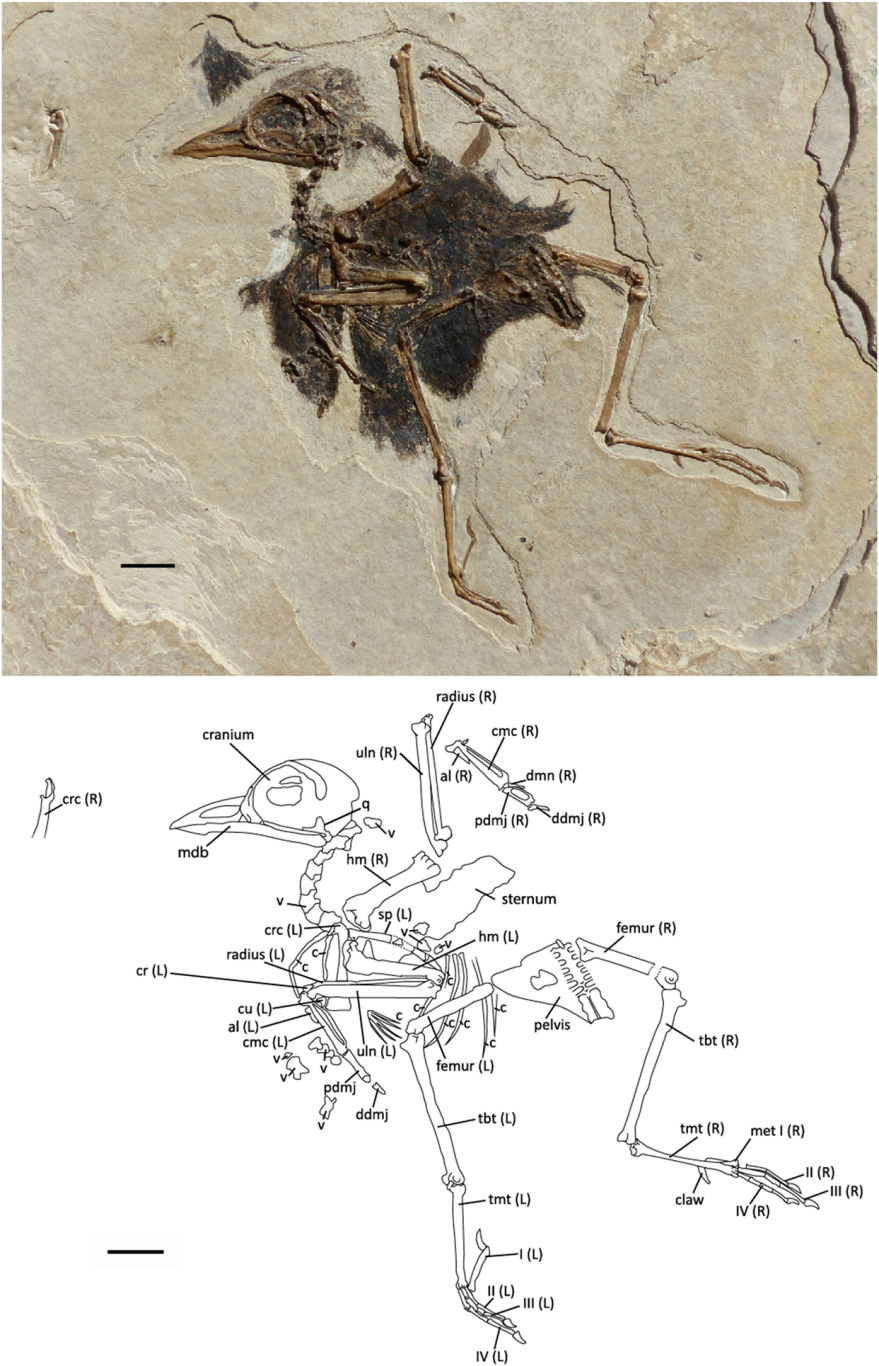
The fossil specimen NT-LBR-014 from Revest-des-Brousses, Luberon (France), and interpretative drawing. al, wing phalanx digiti alulae; c, costa; cmc, carpometacarpus; cr, os carpi radiale; crc, coracoid; cu, os carpi ulnare; ddmj, distal wing phalanx digiti majoris; dmn, wing phalanx digiti minoris; hm, humerus; mdb, mandible; met, metacarpal; pdmj, proximal wing phalanx digiti majoris; q, quadrate; sp, scapula; tbt, tibiotarsus; tmt, tarsometatarsus; uln, ulna; v, vertebra; II, III, IV, numbering of pedal digits. Scale bars, 10 mm.

**Figure 2:**
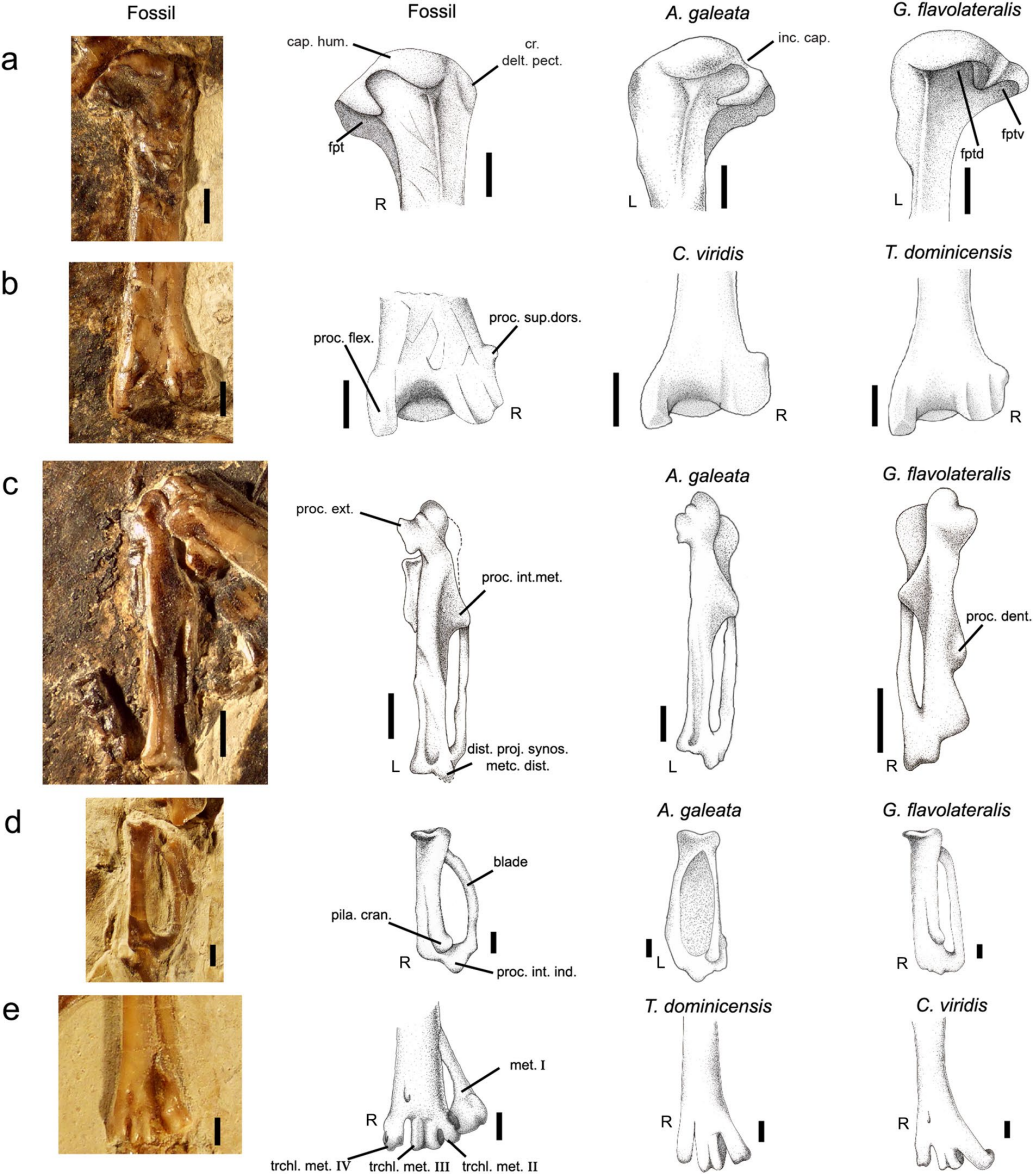
Selected bones of the Luberon fossil NT-LBR-014, compared with extant species (Acanthizidae, Calyptomenidae, Tyrannidae, Pipridae). Second column from the left, drawings of bones which photos are in the first column. Line a, proximal humeri in caudal view. Line b, distal humeri in caudal view. Line c, carpometacarpi in dorsal view. Line d, proximal wing phalanges digiti majoris in ventral view. Line e, distal tarsometatarsi in dorsal view. cap. hum., caput humeri; cr. delt. pect., crista deltopectoralis; dist. proj. synos. metc. dist., distal projection of synostosis metacarpalis distalis; fpt, fossa pneumotricipitalis; fptd, fossa pneumotricipitalis dorsalis; fptv, fossa pneumotricipitalis ventralis; inc. cap., incisura capitis; L, left side; met., metacarpal; pila cran., pila cranialis; proc. dent., processus dentiformis; proc. ext. processus extensorius; proc. flex., processus flexorius; proc. int. ind., processus internus indicis; proc. int. met., processus intermetacarpalis; proc. sup. dors., processus supracondylaris dorsalis; R, right side; trchl. met., trochlea metatarsi. Scale bars, 2 mm (**a**–**c**), 1 mm (**d**,**e**).

The whole morphology of the specimen NT-LBR-014 (Fig. 1), from the early Oligocene of Revest-des-Brousses (Luberon, Alpes-de-Haute-Provence, France), indicates that it belongs to the Passeriformes, to the exclusion of other birds. Among the more distinctive passerine characters, the fossil exhibits (i) trochleae II, III and IV of the tarsometatarsus situated in one plane, and the distal extremities of which are aligned (Figs. 1 and 2); (ii) a carpometacarpus with a wide processus intermetacarpalis (Fig. 2), a character found outside Passeriformes only in the Galliformes, Piciformes, Coliiformes and Coraciiformes (which differ from passerines by other characters)^18^; and (iii) the processus intermetacarpalis and the os metacarpale minus are fused in the fossil, which is found only in Passeriformes^21^ and Piciformes^22^, the latter differing in many other characters (among which zygodactylous type tarsometatarsus trochleae). Among numerous other passerine characters, the fossil also exhibits a tibiotarsus with two equally-sized and parallel condyles, not curved laterally or medially.

### Assignment to the Eupasseres (Passeri and Tyranni)

Acanthisittids are osteologically very derived, probably owing to their long insular isolation without predators, which favoured characters associated with reduction of flight ability, apparent in certain species (some are or were even flightless). The fossil differs from acanthisittids by numerous characters (Supplementary Table 1). Among these characters, the acanthisittid humerus is more curved (S-shaped), a shape approached by the Rhinocryptidae, also poorly flighted, contrary to the fossil which exhibits a straight humerus (Fig. 1). In addition, the fossa pneumotricipitalis is double in acanthisittids, as in most Passeri, whereas it is unique in the fossil (Fig. 2). The combination of those two characters is found only in acanthisittids.

In addition, among characters less susceptible to be associated with flight reduction (see also cranial characters in Supplementary Table 1), the coracoid in acanthisittids has a shape much different from that of the fossil and the extant Eupasseres, and does not possess a foramen situated medially at the base of the processus acrocoracoideus (present in the extant Eupasseres and the fossil). The acanthisittid carpometacarpus also exhibits differences, notably a more proximally situated processus intermetacarpalis, compared with the fossil and other passerines. The fossil therefore differs from the Acanthisittidae, and belongs to the Eupasseres.

### Assignment to the tyranni

**Figure 3:**
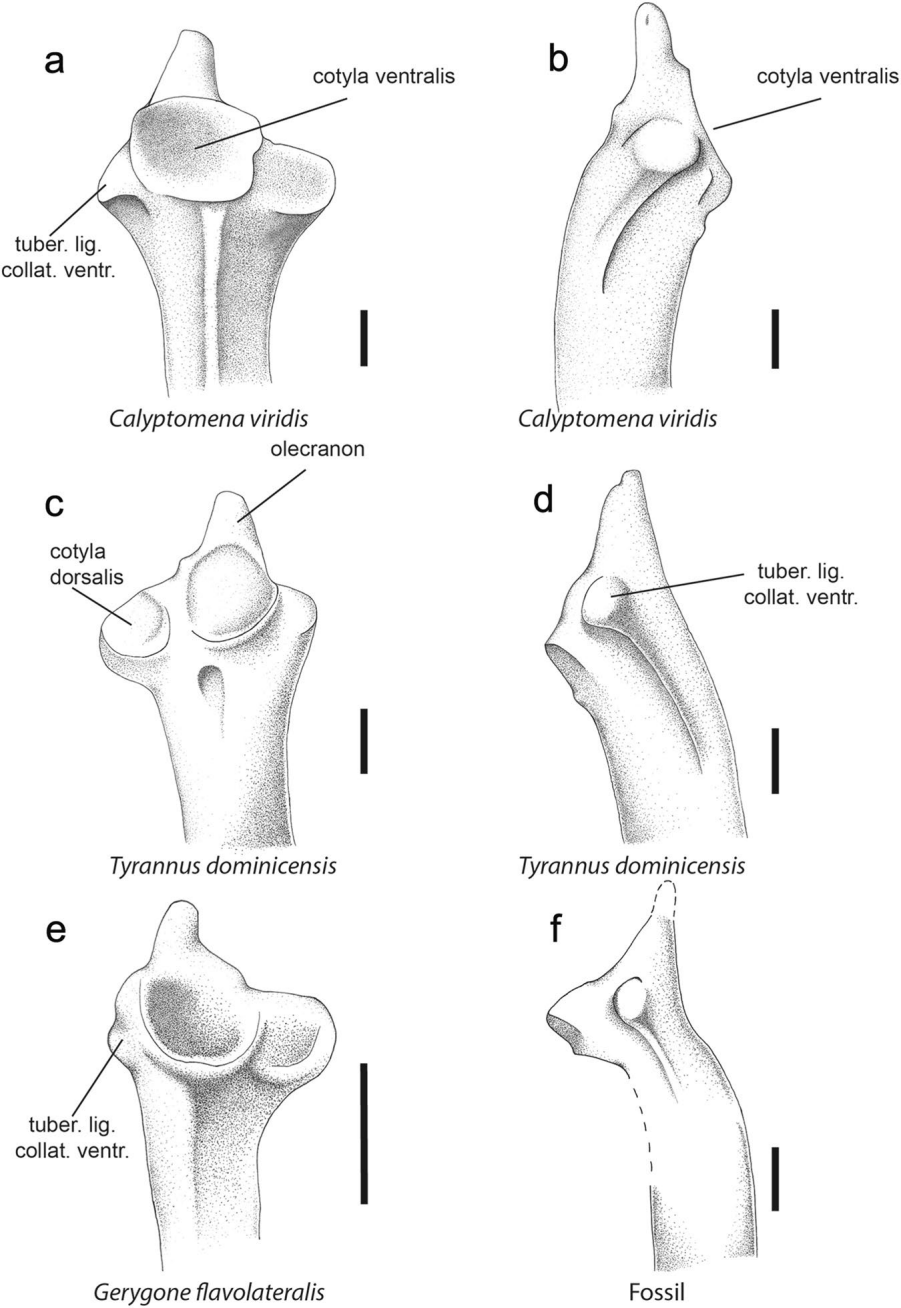
Drawings of right ulna of the Luberon fossil NT-LBR-014 and ulnas of extant species (Passeri, Acanthizidae; Tyranni, Calyptomenidae and Tyrannidae). (**a**,**b**,**e**), left ulnas; (**c**,**d**,**f**), right ulnas; (**a**,**c**,**e**), cranial views; (**b**,**d**,**f**), ventral views. tuber. lig. collat. ventr., tuberositas ligamenti collateralis ventralis. Scale bars, 1 mm.

The wing elements are especially diagnostic for differentiation between the two sub-orders^5,7,8,13^ (Figs. 2 and 3, Supplementary Table 1), but other features are also helpful. These diagnostic features are confirmed, or one yielded, by the present comparative study. As in the Tyranni, the fossil exhibits: a quadrate-quadratojugal articulation of the suboscine type (see ref. ^19^: 136–137.); a prominent tuberculum ligamenti collateralis ventralis of the ulna (little prominent in the Passeri)^13^; a tuberculum carpale more extended and spatulate (vs. shorter and obtuse in the Passeri; new described character); a processus dentiformis of the carpometacarpus poorly individualized (and moderately marked; less marked in some Tyranni; well individualized and strongly marked in the Passeri);^5,7,8,13^ a distal extremity of the os metacarpale minus prominent and pointed (square-shaped, and more hollow in ventral view, in the Passeri);^5,7,8,13^ a blade of the wing phalanx 1 digiti majoris with a rounded, convex border (straight border in the Passeri);^7,19^ presence of a processus internus indicis on the distal extremity of the alar phalanx 1 digiti majoris (absent in the Passeri)^7,19^. These and other diagnostic characters allow to identify NT-LBR-014 as belonging to the Tyranni (and exclude the Passeri).

### Phylogenetic analyses

In order to precise the position of NT-LBR-014 within the Tyranni, phylogenetic analyses in parsimony were conducted based on the distribution of characters across the extant Tyranni examined and the fossil, transformed into a character matrix (Supplementary Methods; strict consensus, Supplementary Fig. 1; bootstrap analysis, Supplementary Fig. 2). The resulting trees show low robustness indices for most nodes (Supplementary Figs. 1, 2). We interpret the low robustness or poor resolution of the trees as the result of pervasive homoplasy in the distribution of osteological character states across the Tyranni (and probably across the whole Passeriformes). This had been observed in previous analyses involving fossil passerines, leading authors to refrain applying cladistic analyses (or other phylogenetic methods) to such osteological datasets^12–14^. Results of our temptative phylogenetic analyses are not incongruent with our more qualitative results below, although they do not offer significant weight per se. *Sapayoa aenigma* is correctly placed in a clade exclusively with other Eurylaimides in the tree generated by bootstrap analysis (1000 replicates), although with poor support (Supplementary Fig. 2), as well as in the strict consensus tree (Supplementary Fig. 1). NT-LBR-014 is found distant from *Sapayoa* (and other Eurylaimides) in both analyses, and in addition it is found in a clade exclusively with piprid taxa in the strict consensus tree. The phylogenetic trees do not make it possible to ascertain which characters are plesiomorphic for the Tyrannida, for example, or synapomorphic for diverse subclades.

In spite of the limitations of phylogenetic analyses based on a character matrix, the distribution of characteristics observed makes it possible, nevertheless, to identify sets of characters that successively exclude taxa in the assignment of NT-LBR-014, and restrain the clade to which it belongs, starting again at the level of the Tyranni.

### Assignment to the Tyrannides

The Tyranni comprises two infra-orders: the Eurylaimides (“Old World Tyranni”) and the Tyrannides (“New World Tyranni”), based on molecular data^4,23–25^. Few skeletal diagnostic characters make it possible to differentiate systematically between members of the two clades. Two of these characters apply to all the Eurylaimides and Tyrannides examined. As in the Tyrannides, the fossil exhibits: a straight processus flexorius of the distal humerus (partly produced, and somehow hooked more dorsally and caudally in the Eurylaimides); a cotyla ventralis of the proximal ulna slightly rounded and little developped ventrally (more rounded and developped ventrally in the Eurylaimides) (Figs. 2 and 3, Supplementary Table 1).

**Figure 4:**
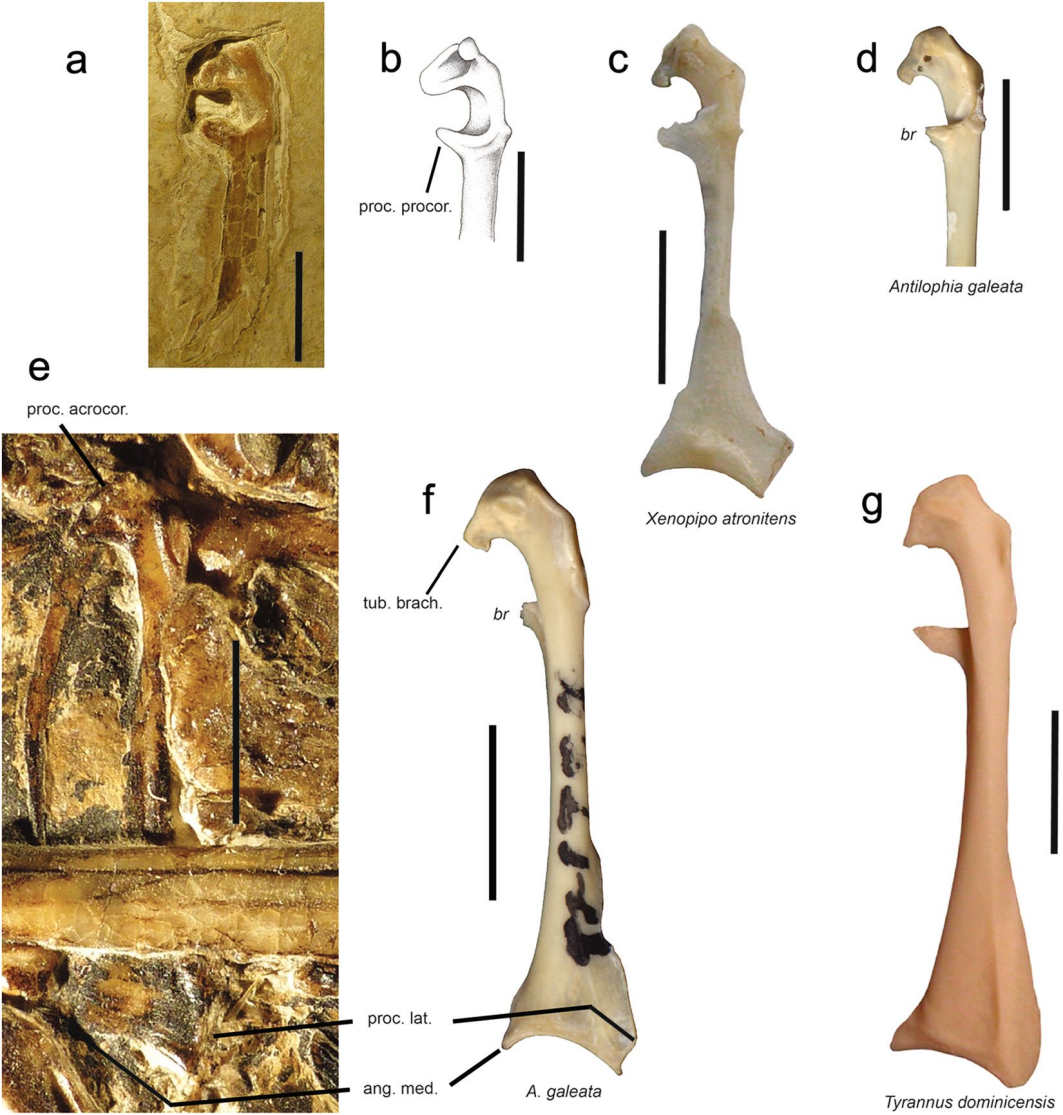
Coracoids of the Luberon fossil NT-LBR-014, compared with extant species (Tyrannida). (**a**,**e**), respectively right (dorsal view) and left (ventral view) coracoids of the fossil NT-LBR-014; b, drawing of (**a**,**c**), dorsal view, inverted left coracoid; (**d**), dorsal view; (**f**), ventral view; (**g**), ventral view, inverted right coracoid. ang. med., angulus medialis; proc. acrocor., processus acrocoracoideus; proc. lat., processus lateralis; proc. procor., processus procoracoideus; tub. brach., tuberculum brachiale; *br*, broken. Scale bars, 5 mm.

In addition, most of the Tyrannides, as well as the fossil, exhibit other characters distinct from the Eurylaimides: more rounded orbits; a brachial tuberosity of coracoid (tuberculum brachiale; Fig. 4) more developed medially; and a processus extensorius of carpometacarpus (Fig. 2) less deported ventrally and slightly laterally. Species in the Tyrannides that exhibit intermediate states for these characters (between Eurylaimides and typical Tyrannides) are: *Geositta cunicularia* (Furnariidae), *Scytalopus unicolor* (Rhinocryptidae), *Formicarius analis* (Formicariidae), *Cotinga* sp. (Cotingidae), *Tyrannus dominicensis* (Tyrannidae). These few intermediate cases do not affect the observation that for all these characters NT-LBR-014 corresponds to the Tyrannides and differs from the Eurylaimides.

### Assignment within the Tyrannides

**Figure 5:**
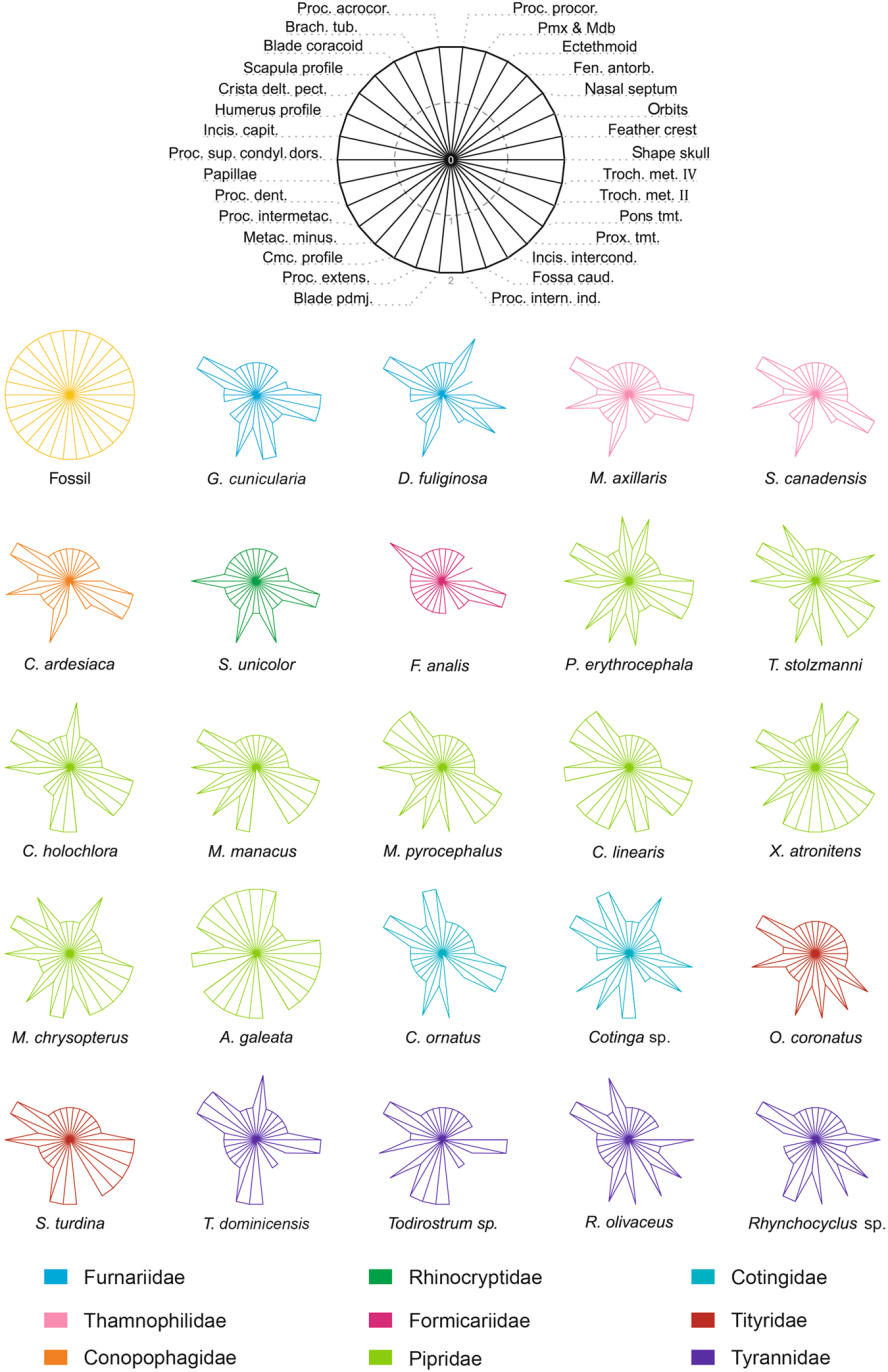
Radial visualisation (Kiviat diagram) of the distribution of character states of the Luberon fossil NT-LBR-014, across extant taxa of the Tyrannides for which all the characters were assessable. Top, position of the diagnostic characters considered here relative to each radius. In extant species, character state can be 0 (centre; character absent), 1 (mid-radius; character present but state still different from fossil), or 2 (state identical or similar to fossil) (see Supplementary Table 4).

Among all characters observed on the fossil, 53 are discriminant among the examined Tyranni, within which many are rather variable across the Tyrannides (Fig. 5, Supplementary Tables 1, 2) and do not help link the fossil with a particular family or genus. Nevertheless, a number of other characters on each skeletal element appear diagnostic for one or several families (Supplementary Table 1); the more prominent ones are detailed below.

### Skull

**Figure 6:**
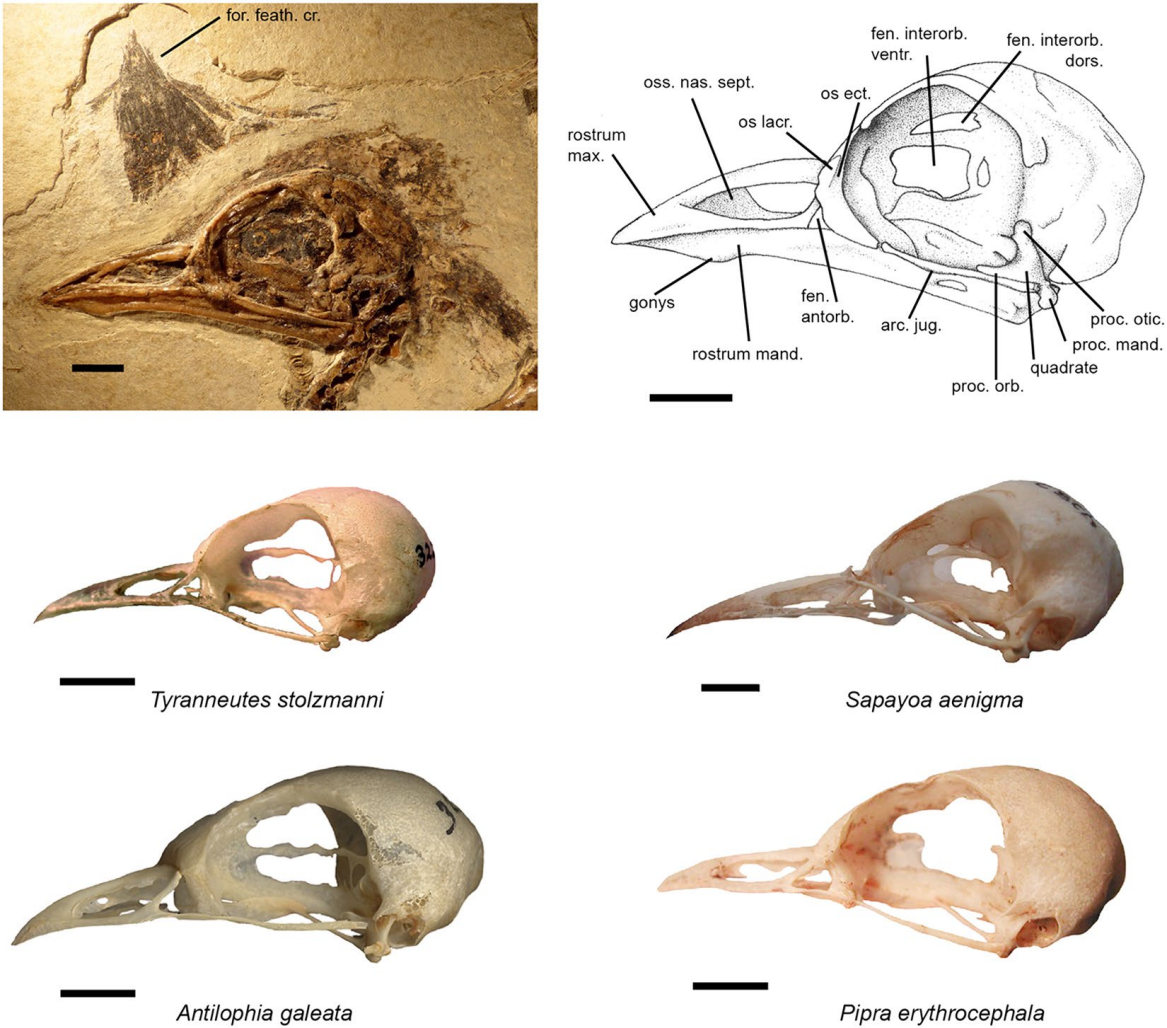
Skull of the Luberon fossil NT-LBR-014, compared with extant species (Pipridae, Sapayoidae). All left lateral views. Top left, fossil NT-LBR-014; top right, drawing of the preceding. NB: the part below the os lacrimale is collapsed, and seems to have been developed as in, e.g., *Sapayoa*. arc. jug., arcus jugalis; fen. antorb., fenestra antorbitalis; fen. interorb. dors., fenestra interorbitalis dorsalis; fen. interorb. ventr., fenestra interorbitalis ventralis; for. feath. cr., forehead feather crest; os ect., os ectethmoidale; os lacr., os lacrimale; oss. nas. sept., osseous nasal septum; proc. orb., processus orbitalis of quadrate; proc. otic., processus oticus of quadrate; proc. mand., processus mandibularis of quadrate; rostrum mand., rostrum mandibulare; rostrum max., rostrum maxillare. Scale bars, 5 mm.

The fossil, as well as *Xenopipo atronitens* (Pipridae), exhibit a reduced and triangular anteorbital fenestra (Fig. 6, Supplementary Table 1). The other species exhibit a fenestra generally more developed proportionally, and less neatly triangular.

The dorsal interorbital fenestra is smaller than the ventral, and the separation between them is thin, in the fossil (Fig. 6, Supplementary Table 1). The relative size of these fenestrae shows great variability across extant species and families. However, there are certain trends in the position of these fenestrae, relative to the orbit, between families. The fenestrae generally start rostrally at the same level relative to the orbit, in species of a given family. In the fossil, the rostral extremity of the fenestrae lies at the rostral ¼ of the orbit length, like in the Pipridae.

The outline of the cranium, orbits, (and beak) of the fossil, in comparison with extant Tyrannides, also helps delimiting close similarities of several characters with different taxa: one genus in the Tityridae and two in the Tyrannidae, but several in the Pipridae, and also *Sapayoa* (Sapayoidae), different suites of characters being involved for every of these taxa (Supplementary Table 1, Fig. 6). Incidentally, among piprid taxa, for *Antilophia*, which otherwise shares a number of similarities with NT-LBR-014, differences mainly concern a few cranial characters (Fig. 5); another piprid, *Neopelma*, is in contrast similar to the fossil in most cranial characters (including the marked gonys of mandible), and less so in postcranial ones.

**Figure 7:**
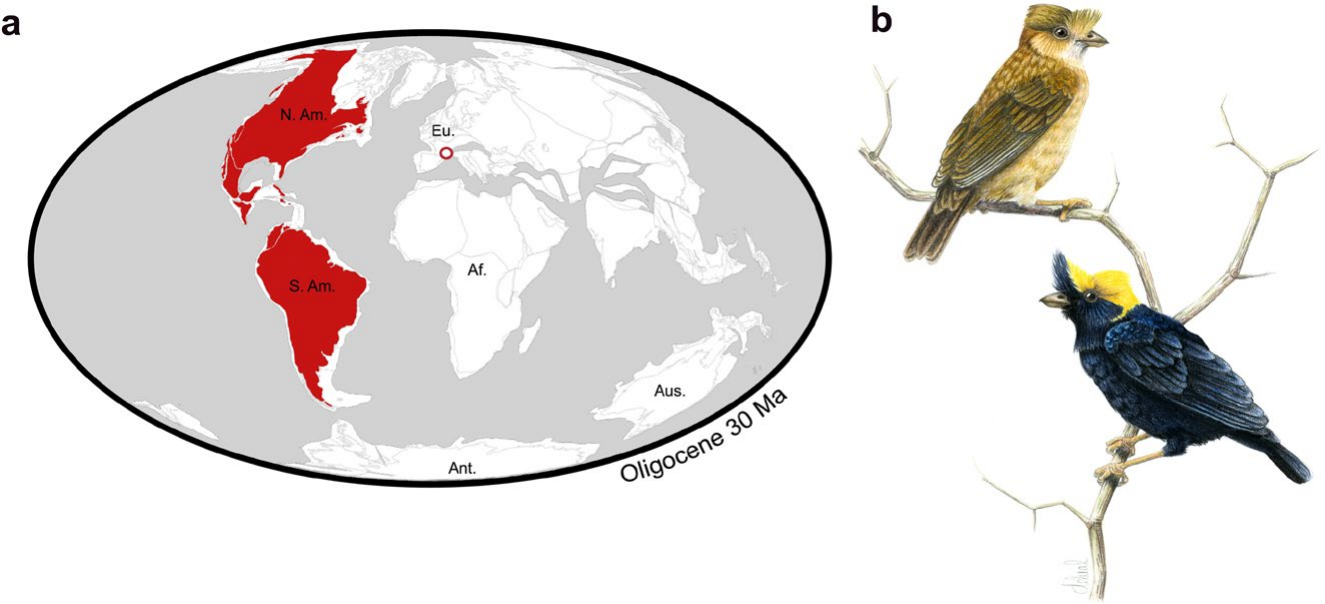
Geographic location, and reconstruction of the Luberon early Oligocene Tyrannida. In (**a**), the geographic location of the Luberon Tyrannida NT-LBR-014 is represented (red circle), together with the extant distribution of Tyrannida (red area), superimposed on a paleogeographic map of landmasses in the early Oligocene (map background modified after The Paleobiology Database). In (**b**), reconstruction of the Luberon Oligocene manakin-like Tyrannida in life; drawing copyright Manon Delval.

The feather crest erected above the rostrum basis of NT-LBR-014, in close examination, is clearly in exact life position and shape, and has been unaffected by taphonomic processes. It is triangular, well-developed, directed rostrally but with the tip slightly recurved caudally (Figs. 1 and 6, Supplementary Table 1). Several families comprise species that exhibit a crest (or crests) on the head, but different in shape and/or in precise position (Tyrannidae, Tityridae, Cotingidae, Thamnophilidae, Rhinocryptidae, Furnariidae). Only in certain Pipridae a crest above the beak exhibits a shape approaching (*Chiroxiphia*, *Masius*) or being identical (*Antilophia*) to that of the fossil. The crest of the fossil is only slightly larger proportionally (18.6 mm length) than that of *A. galeata* (13.0–14.5 mm), with a coefficient of proportionality of ca. 4/3 (see Fig. 7B).

### Coracoid

The fossil coracoid exhibits a prominent processus acrocoracoideus (Fig. 4), with a shape similar to that of *A. galeata* (Pipridae).

The processus procoracoideus of the fossil is well-developed medially (Fig. 4), and is similar to that of *Tyrannus dominicensis* (Tyrannidae). This process has a shape approaching that of the piprid species *C. holochlora* and *X. atronitens* at least (broken in the available specimen of *A. galeata*, also suggesting prominent shape).

### Humerus

*Scytalopus unicolor* (Rhinocryptidae) differs from other extant taxa examined and the fossil by the reduced crista deltopectoralis, a character linked with reduced flight capability^26^. The fossil exhibits a processus supracondylaris dorsalis that is unique and well-developed (Fig. 2), a character shared with all the Pipridae, and *Pitta sordida* (Pittidae), *Sapayoa aenigma* (Sapayoidae), *Scytalopus unicolor* (Rhinocryptidae), *Schiffornis turdina* (Tityridae), and *Pipreola arcuata* (Cotingidae); the other extant species examined in the Tyrannides have a unique processus supracondylaris dorsalis, but which is reduced (or less prominent proximally).

### Ulna

The fossil exhibits relatively reduced papillae remigales caudales, similar to the condition in *Myrmotherula axillaris* (Thamnophilidae), *Conopophaga ardesiaca* (Conopophagidae), *Oxyuncus cristatus* (Tityridae), *Todirostrum* sp. (Tyrannidae), and most of the Pipridae.

### Carpometacarpus

**Figure 8:**
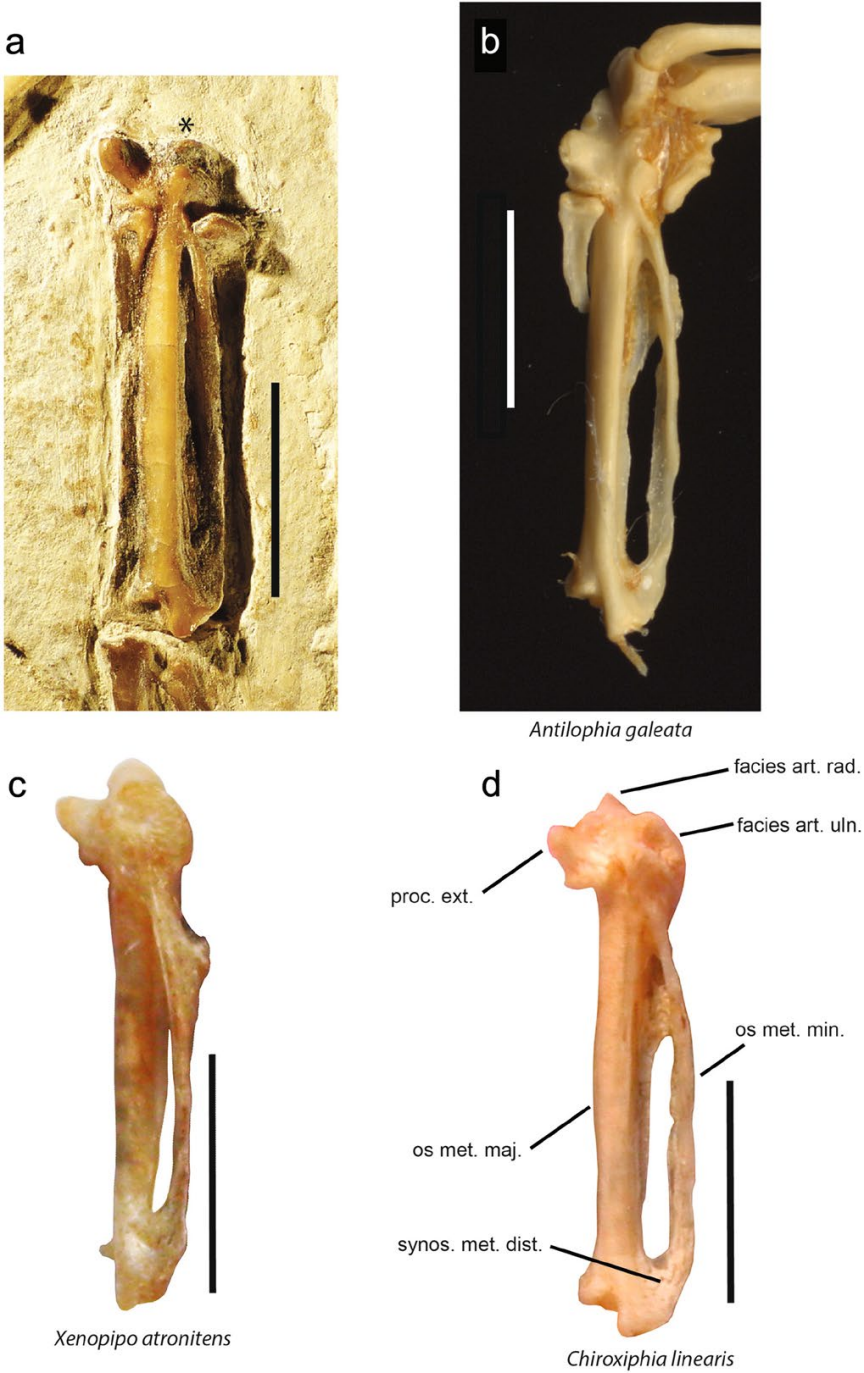
Right carpometacarpus of the Luberon fossil NT-LBR-014, compared with extant species (Pipridae). (**b**,**c**), inverted left carpometacarpi. In (**c**), the angle of view (ventral) is slightly different from that in (**a**,**b**,**d**) (slightly cranio-ventral, in (**b**) more than in (**a**,**d**)). *The facies articularis radiocarpalis is masked under the matrix. facies art. rad., facies articularis radialis; facies art. uln., facies articularis ulnaris; os met. maj., os metacarpale majus; os met. min., os metacarpale minus; proc. ext., processus extensorius; synos. met. dist., synostosis metacarpalis distalis. The angles of view prevent from seeing the processus dentiformis when present. Scale bars, 5 mm.

The shape and position of the processus intermetacarpalis in the fossil are similar to those observed in *C. linearis*, *X. atronitens*, *A. galeata* (Pipridae), and *Cotinga* sp. (Cotingidae) (Fig. 2). The processus dentiformis in NT-LBR-014 is well marked, as is observed in some taxa of the Eurylaimides (including *Sapayoa*), as well as some Conopophagidae, Rhinocryptidae, Formicariidae and Pipridae in the Tyrannides. In the Pipridae, a marked processus dentiformis is seen in *Manacus* and *Xenopipo*. The outline of the bone is otherwise similar to that in several piprid species (Fig. 8).

### Wing phalanx digiti majoris 1

Among the Tyrannides, certain families exhibit a processus internus indicis that is only faint, or even absent: the Furnariidae, Thamnophilidae, Conopophagidae, Rhinocryptidae, Formicariidae, Dendrocolaptidae. In addition, in these families the shape of the blade is intermediate between the typical Passeri state (straight border) and the typical Tyranni state (convex border), a character directly linked with the development of the processus internus indicis^19^. These six families can therefore be differentiated from the other Tyrannides and the fossil NT-LBR-014 (Fig. 2) based on these characters.

### Femur

**Figure 9:**
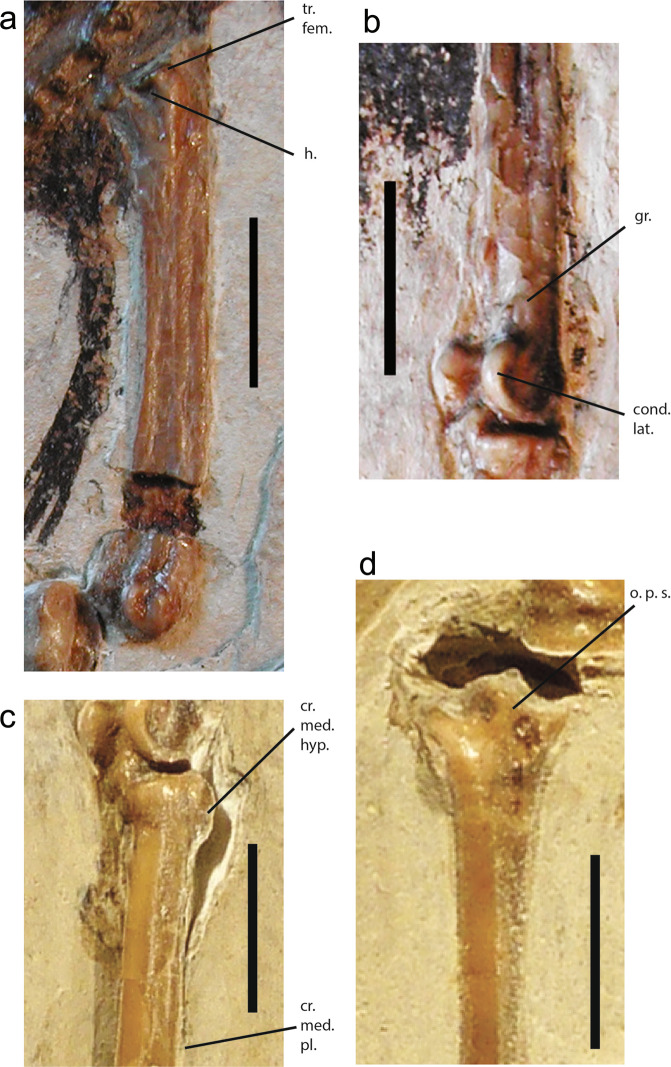
Leg bones of the Luberon fossil NT-LBR-014. a, right femur, latero-caudal view; b, left distal tibiotarsus, latero-cranial view; c, left proximal tarsometatarsus, latero-dorsal view; d, right proximal tarsometatarsus, dorsal view. cond. lat., condylus lateralis; cr. med. hyp., crista medialis hypotarsi; cr. med. pl., crista medialis plantaris; gr., tiny groove between the tuberositas retinaculi extensorius lateralis and the tuberculum retinaculi m. fibularis, proximal to the condylus lateralis (see Supplementary Table 1); h., hollow just distal to the facies articularis antitrochanterica; o. p. s., ossified pons supratendineus; tr. fem., trochanter femoris. Scale bars, 5 mm.

The proximal end of the fossil femur exhibits a rather deep caudal fossa (Fig. 9), a character observed in *Geositta cunicularia* (Furnariidae), *Scytalopus unicolor* (Rhinocryptidae), and *Masius chrysopterus*, *C. linearis* and *X. atronitens* (Pipridae).

### Tibiotarsus

A medial crest on the proximal end is absent, contrary to *Scytalopus unicolor* (Rhinocryptidae), *Phytotoma rara* (Cotingidae), *Rhynchocyclus olivaceus* (Tyrannidae), and most of the Tityridae examined, which exhibit a marked crest.

### Tarsometatarsus

The fossil exhibits an ossified pons supratendineus on the proximal part of the dorsal face, positioned rather proximally. A pons is positively absent in only two of the examined extant passerines, *Tyrannus dominicensis* and *Todirostrum* sp. (both Tyrannidae), and at least no other Tyranni (Fig. 9, Supplementary Table 1).

### Combinations and distributions of characters

A Kiviat diagram allows visualization of the distribution of the states of the characters that show heterogeneity across extant Tyrannides, and the states observed in the fossil (Fig. 5). The families in the Tyrannides are grouped into two clades: the Tyrannida and the Furnariida. A number of characters are shared between the fossil and the Tyrannidae, Cotingidae, Tityridae and Pipridae –these four families forming the Tyrannida– and differ from those in the Furnariida. In addition, all the characters of the fossil are similar to those of at least one of the examined species of Pipridae. And last, the fossil shows a greater resemblance overall with *C. linearis* and *X. atronitens*, and above all a maximum of similar/identical characters with *A. galeata*.

NT-LBR-014 exhibits a mosaic of characters present in one or more families of Tyrannides, and systematically in some or all of the examined Pipridae, contrary to other families (Fig. 5, Supplementary Table 1, and also Supplementary Table 2 showing six additional characters that are discriminant for certain genera and species across the Tyrannida).

## Discussion

### Systematic assignment of the Oligocene fossil

NT-LBR-014, unambigously assignable to the Tyranni within the Passeriformes, can also be firmly placed more precisely in the Tyrannides. All the character states of NT-LBR-014 are systematically present in members of the Tyrannides and they include the diagnostic characters of Tyrannides, to the exclusion of the Eurylaimides, that we highlighted. Eventhough with poor support, our temptative phylogenetic analyses are concordant with this result, placing the fossil outside the Eurylaimides, the latter comprising *Sapayoa* in agreement with molecular works (see below). Within the Tyrannides, several characters exclude the infra-order Furnariida (composed of the Furnariidae –this family including the former Dendrocolaptidae^1^, the Thamnophilidae, Conopophagidae, Formicariidae, and Rhinocryptidae)^1,2,27^, and no character state is shared only between the fossil and one or more members of these six families to the exclusion of other Tyrannides –the Tyrannida. Not all the extant genera (not to mention species) could be examined in the Furnariida, but a sample that we consider sufficiently well-distributed phylogenetically, to allow for some extrapolation of the character states that were observed, and which differ systematically from NT-LBR-014. The most genus and species-rich families in the Furnariida are the Thamnophilidae and the Furnariidae. The representatives examined (or for which data are available in the literature) are considered sufficiently different from the fossil to be confident in our conclusions. Conversely, most characters are shared between the fossil and the other infra-order, the Tyrannida (composed of the families Cotingidae, Tityridae, Tyrannidae, and Pipridae^1,2,27^). Specimens of Cotingidae, Tityridae and Tyrannidae differ from NT-LBR-014 mostly in characters of the skull and the coracoid. The fossil shares a maximum of characters with the Pipridae, and every character is in common with at least one, or all of the genera examined in the Pipridae. Similarity is greater with the Piprinae (*Chloropipo*, *Antilophia*, *Chiroxiphia*, *Masius*, *Xenopipo*, *Manacus*, *Pipra*, *Machaeropterus*) on postcranial characters, and with the Neopelminae (*Neopelma* et *Tyranneutes*; the more basal subfamily of Pipridae^28^) on cranial characters (principally with *Neopelma*). Within the Piprinae, the fossil shares a greater number of postcranial characters with *Chiroxiphia* and *Xenopipo*, and an even greater number with *Antilophia* (26 of 30 characters, excluding those diagnostic for Passeriformes and for Tyranni). However, NT-LBR-014 exhibits a mosaic of characters present in several different piprid genera (Piprinae or Neopelminae; Supplementary Table 1). But moreover, it is possible that osteological characters are also shared with at least one other Tyrannida outside the Pipridae. This is especially possible in the family Tyrannidae since a number of extant genera and species could not be examined among the 449 species in 101 genera of this extremely rich family. Among the 67 species in 24 genera of Cotingidae, or the 49 species in 11 genera of Tityridae, most could not be seen either. As a consequence, some characters here found in common exclusively with some piprid taxa could possibly be plesiomorphic for the Pipridae, or even plesiomorphic for the Tyrannida as a whole, and present also in other families. Some of the Tyrannidae examined already show a number of shared characteristics with the fossil, although less than the piprid taxa. Even rare features such as the particular feather crest could be found in an Oligocene fossil through plesiomorphy or convergence outside crown Pipridae, or even in another family. Therefore, even if more extant species of Tyrannida were examined and considered here than in all previous literature on an early fossil passerine, we suggest, pending a more thorough survey of other taxa in the Tyrannida, to conservatively assign NT-LBR-014 to the Tyrannida, more probably as a stem representative.

Interestingly, a synamoporphy of Pipridae has been known since the 19th century, namely the syndactyly of the outer toes (III and IV); and this character also evolved convergently in some members of other clades in the Tyranni^29^. Aware of this character, we nevertheless found no indication of fusion between phalangeal bones themselves, in any extant piprid, nor in any other extant specimen examined (Supplementary Fig. 3). The syndactyly of toes III and IV in Pipridae, as well as other forms of syndactyly, were observed exclusively on naturalized specimens^29^, and obviously they concern only the soft tissues surrounded the bones. Therefore, the absence of fusion of toe bones in the fossil (Supplementary Fig. 3), as well as on all extant specimens examined, has no bearing on reported syndactyly, wich rests on soft tissues, and the latter is a character out of reach on the fossil.

The early assignment of *Sapayoa* to the Pipridae in the history of classification, on the basis of morphology, is consistent with the osteological partial resemblance on some characters noticed here between these two taxa. More recently, molecular phylogenetic analyses revealed that *Sapayoa* belonged in the Eurylaimides, of which it is the only New World representative^2,3,30^. The characters of *Sapayoa* showing similarity with the Pipridae, as well as with the fossil, are therefore interpretable as the result of convergences.

NT-LBR-014 shows no close similarity with the few incomplete passeriform fossils found in the Oligocene or early Miocene of France, Germany and Poland^5–15,31^, including a nearly completely represented taxon from the early Oligocene of Germany, *Wieslochia weissi*^12,13^ (Supplementary Table 1), which displays a greater number of assessable characters than others. Although disarticulated and with moderately well preserved detail, *W. weissi* exhibited features leading to consideration of its position as probably basal in the Tyranni, or Eupasseres, or even Passeriformes as a whole^13^. Incidentally, a range of comparable phylogenetic positions (including within crown passerines) is indeed plausible for some European Miocene tarsometatarsi, the hypotarsus of which had initially led Manegold *et al*^31^., to consider them outside crown Passeriformes^32^.

### Paleoecology

With a length of 15 cm, the fossil NT-LBR-014 is a medium-sized Tyrannida; its legs are of medium length proportionally, as well as the wings (Supplementary Table 3, Supplementary Fig. 4). The beak and claw shapes are also unspecialized compared with modern Tyrannida, and are compatible with a rather generalist diet, comprising insects and small fruits, as in most extant manakins, tyrant-flycatchers and allies. Extant Tyrannida live in the Americas, with most diversity in the neotropical ecozone^1^. NT-LBR-014 derives from a near-coastal lagunar, freshwater depositional setting, surrounded by forests, under a subtropical to tropical paleoclimate^33–35^, consistent with the ecological requirements of the vast majority of present-day members of the Tyrannida; only the Tyrannidae expand across entire North America in the breeding season, in addition to the Neotropics.

### Early passerines and molecular ages

Recent molecular studies have determined the age of divergence between Acanthisittidae and Eupasseres (Passeri and Tyranni) as around the Paleocene-Eocene limit (ca. 56 Ma)^3^, or later in the early Eocene, near 48 Ma^4^. The earliest ascertained fossil passerines are from the early Oligocene of Europe. They comprise Passeri, Tyranni and possibly more basal lineage(s)^5–15^. NT-LBR-014 is the first to be assignable to a more precise, extant passerine clade, the Tyrannida, at ca 30 Ma. A molecular age of diversification for the Tyrannida was proposed at 32–33 Ma^2,27^ or near 24 Ma^4^, and the divergence between Tyrannida and Furnariida at 38.9 Ma^3^ or near 36 Ma^4^. The identification of NT-LBR-014 as a stem Tyrannida, or possibly situated at the start of the diversification of the Tyrannida, is congruent, at ca 30 Ma, with these molecular results. Furthermore, this fossil will now offer a new calibration point for a minimal age of stem Tyrannida (prior to crown diversification), for future molecular studies, which would presumably tend to slightly increase the diversification ages cited above.

### Paleobiogeography of the Tyranni

The early Oligocene presence in Europe of a Tyrannida, a clade today exclusively American (Fig. 7), might be explained by several different scenarios, as for two other stem-representatives of New World clades found in the same area: the stem hummingbird *Eurotrochilus* sp.^34^ and the stem Galbulae *Jacamatia*^36^. The stem Tyrannida may have originated in the New World, and then the presence of a Tyrannida in southern France in the Oligocene implies that they rapidly colonized Europe in the early Oligocene. This passage might have taken the route of landmasses and straits between northern North America and Europe. Fossil records of Tyrannida (and other Tyranni) are lacking in Oligo-Miocene or older strata of northern America to support this hypothesis, but this apparent absence does not rule out the hypothesis since sufficiently diagnostic fossil passerines are extremely rare worldwide in these periods in general. The passage might alternatively have been from southern America to Europe, directly or via Africa, where the avian fossil record is extremely scarce for these periods. Alternatively, the stem Tyrannida may have originated in the Old World. A new fossil such as NT-LBR-014 can disrupt models that are inferred^4^ based only on extant distributions. As is the case for the stem hummingbirds^34^ and stem Galbulae^36^ found in Europe in the early Oligocene, the new fossil Tyrannida calls for the possibility of a much more complex history of past distributions. In the hypothesis of an Old World origin of stem Tyrannida (and hence, probably also the stem Tyrannides, from the Old World stem of its sister clade Eurylaimides), they must have colonized the Americas at some point between the early Oligocene and the middle Miocene. Again, the passage could have occurred north of the northern Atlantic via northern America, or from Europe to southern America. In the latter case, an additional scenario might be envisioned as colonisation of southern America by European populations becoming medium-distance or long-distance seasonal migrants, in a context of increased seasonality during these periods^37^. Such populations would have been progressively wintering in southern America where descendents would have become more resident later in evolution. In both scenarios of family origin, transatlantic crossing by the northern route was rendered possible by the tropical to subtropical climate up to high latitudes, but preferentially early in the Oligocene, owing to later global cooling stages^38^. And in both scenarios, crossing between northwest Africa and South America would have required a transit of “only” 1,000 km across the ocean, and progressively more with continental drift. Paleo-islands in the southern Atlantic in the Oligocene^39^ would have helped this crossing. A last possibility of passage would have been via the Bering Strait which benefited from a mild climate, but the absence of fossil evidence added to the much greater distance, make this scenario much less likely.

In every hypothesis, after the Oligocene the European distribution of Tyrannida would have become reduced and eventually disappeared at latest in the upper Miocene, owing to global cooling and a decrease in winter temperatures among other factors^37,38^. This led to a relictual distribution in the southern hemisphere, tropical regions, in this case neotropical zones, as was the case for several other bird groups^36,40,41^. Concomitant with this retreat towards the equator in America, some lineages could become progressively long-distance migrants (including members of the Tyrannidae today breeding in North America and wintering in the Neotropics). Interestingly, *Sapayoa aenigma*, “Old World” Tyranni (Eurylaimides) living in South America, also illustrates a transatlantic crossing of an ancestor, leaving descendents on both sides (this species is neotropical, and all other Eurylaimides are paleotropical). It is not possible to favour a northern or a southern passage in the case of *Sapayoa*, but it must have occurred between the latest Oligocene and middle Miocene^3^.

## Methods

### Fossil material

The fossil NT-LBR-014 (collection Nicolas Tourment, Marseille^34,35^) is a nearly complete articulated skeleton on slab, embedded in fine limestone laminites. A cast is deposited in the Collections of the Université Lyon 1-Claude Bernard (Villeurbanne, France, collection n° UCBL-FSL-444666). The depositional setting was calm; only a few bones are disarticulated (e.g., the right coracoid is slightly displaced). Parts of the feathering are preserved as a thin layer of dark organic matter, showing among other features the shape of a typical frontal crest, in place and undisturbed. The laminites were deposited in a coastal freshwater to slightly brackish lagoon, and date to the early Oligocene (“Vachères limestones”, Rupelian strata, biozone MP24, 33–28.25 Ma^42–45^) of Revest-des-Brousses (Apt Basin, Luberon, Alpes-de-Haute-Provence, southeastern France). These levels locally comprise elements of a tropical to subtropical fauna and flora, essentially of continental origin, and including birds of a dozen families^34–36^.

### Comparative material

Comparisons were made with representatives of the families osteologically close to passerines, and within passerines with a representative sample of most families in the Passeri, as well as with Acanthisittidae and members of all families of Tyranni (41 species), and also with the literature (extant and fossil taxa) (Supplementary Methods, Supplementary Table 1).

### Comparative anatomy, osteological nomenclature, and systematics

Observations of the fossil and extant specimens were realized using a binocular microscope at various magnifications. Drawings were additionally realized using a camera lucida with binocular microscope. Osteological nomenclature follows primarily Baumel and Witmer^46^, unless stated otherwise. Systematic arrangement follows Del Hoyo *et al*^1^..

### Phylogenetic analyses

Methods used for phylogenetic analyses in parsimony are in Supplementary Methods.

## Supplementary information

Supplementary Information.

## Data Availability

Data analysed during this study are included as Supplementary Information files. The fossil NT-LBR-014 is deposited in the Collection Nicolas Tourment, Marseille, and is accessible upon request. The cast UCBL-FSL-444666 is deposited in the Collections of Paleontology, Université Lyon 1, Villeurbanne. Any additional data are available from the author upon reasonable request.
