# Supplementary material for: The earliest Tyrannida (Aves, Passeriformes), from the Oligocene of France
Source: Sci Rep. 2020 Jun 17;10:9776. doi: 10.1038/s41598-020-66149-9 (PMC7299954; doi:10.1038/s41598-020-66149-9)
Supplement: Supplementary file 1 — Supplementary Information. [file 41598_2020_66149_MOESM1_ESM.pdf]

Supplementary Information

The earliest Tyrannida (Aves, Passeriformes), from the Oligocene of France

Ségolène Riamon, Nicolas Tourment & Antoine Louchart

This PDF file includes:

Supplementary Methods

Supplementary Figures 1 to 4

Supplementary Tables 1 to 5

References (for Supplementary Table 1)

Supplementary Methods

List of extant specimens examined, and their collection acronyms

In addition to preliminary comparisons with representatives of almost all other avian families, including a number of derived Oscine passerines (UCBL), skeletal specimens of the following extant species were examined for comparison. (F, female; M, male).

Acanthisittidae, *Acanthisitta chloris chloris*, MNZ 26466; Pittidae, *Pitta sordida*, LAC 1884.2469; Philepittidae, *Philepitta castanea*, F, FMNH 384764; Eurylaimidae, *Cymbirhynchus macrorhynchos*, LAC 1884.256; Calyptomenidae, *Calyptomena viridis*, LAC 1997.830; Sapayoidae, *Sapayoa aenigma*, M, USNM 428203; Furnariidae, *Geositta cunicularia*, M, USNM 614657; *Dendrocincla fuliginosus*, M, FMNH 321160; Thamnophilidae, *Myrmotherula axillaris*, M, FMNH 319192; *Sakesphorus canadensis loretoyacuensis*, M, FMNH 389206; *Thamnophilus doliatus*, (skull) LAC 1997.556; *Thamnophilus coecus*, (skull) LAC 1884.1557; Conopophagidae, *Conopophaga ardesiaca saturata*, M, FMNH 322380; Rhinocryptidae, *Scytalopus unicolor*, M, USNM 559977; Formicariidae, *Formicarius analis*, M, USNM 612383; Pipridae, *Ceratopipra erythrocephala*, LAC 1880.125; *Neopelma sulphureiventer*, M, MZLSU 101474; *Tyranneutes stolzmanni*, F, FMNH 322555; *Chloropipo holochlora viridior*, M, FMNH 322514; *Manacus manacus trinitatis*, FMNH 394500; *Machaeropterus pyrocephalus pyrocephalus*, M, FMNH 322563; *Chiroxiphia linearis fastuosa*, F, FMNH 434065; *Xenopipo atronitens*, M, USNM 622077; *Masius chrysopterus*, M, MZLSU 89991; *Antilophia galeata*, USNM 321704; Cotingidae, *Pipreola arcuata*, F, MZLSU 104416; *P. intermedia signata*, (skull) LAC 1884.1554; *Cephalopterus ornatus*, LAC 1986.37; *Cotinga* sp., LAC n° 2278; *Procnias* sp., (skull) LAC 1884.1583; *Rupicola rupicola*, LAC 2004.635; *Rupicola* sp., (skull) LAC 1884.1574 (=111/711); *Phytotoma rara*, NHM 1891.7.20.273; Tityridae, *Oxyruncus cristatus*, M, MZLSU 108942; *Onychorhynchus coronatus*, NHM 1891.7.20.143; *Tityra semifasciata*,

NHM 1891.7.20.16; *Schiffornis turdinus amazonus*, F, FMNH 322490; Tyrannidae, *Tyrannus dominicensis*, LAC 1996.60; *Tyrannus* sp., LAC; *Todirostrum* sp., LAC 2000.556; *Contopus latirostris*, (skull) LAC; *Rhynchocyclus olivaceus*, NHM S/1974.11.83; *Rhynchocyclus* sp., LAC 2000.459; Menuridae, *Menura novaeohollandiae*, LAC 1883.2208; Ptilonorhynchidae, *Sericulus* sp. LAC 1845.92, *Ptilonorhynchus violaceus* LAC A 4307 = BVI/417; Meliphagidae, *Meliphaga lewini* LAC 1883.2127 = IV/342, *Philemon corniculatus* LAC A 4319 = BVI/370, *Manorina melanocephala* LAC A 4468 = BVI/364; Pardalotidae, *Pardalotus punctatus* LAC 1860.96; Acanthizidae, *Gerygone flavolateralis*, LAC 1997.535; Pomatostomatidae, *Pomatostomus temporalis trivirgatus*, LAC A 4467 = BVI/373; Paradisaeidae, *Ptiloris paradiseus* LAC 1860.102 = A 4291 = BVI/360, LAC A 4295 = BVI/359, *Paradisaea minor* LAC 1878.620.

Institutions acronyms

FMNH, Field Museum of Natural History (Chicago, USA); LAC, Laboratoire d'Anatomie Comparée (Muséum National d'Histoire Naturelle, Paris, France); NHM, Natural History Museum (Tring, UK); MNZ, Museum of New Zealand Te Papa Tongarewa (Wellington, New-Zealand); MZLSU, Museum of Zoology, Louisiana State University (Baton Rouge, USA); UCBL, Université Claude Bernard Lyon 1 (Villeurbanne, France); USNM, National Museum of Natural History (Smithsonian Institution, Washington, D.C., USA).

Methods for phylogenetic analyses

The phylogenetic analyses were realized using 36 characters, which were discriminant for at least one among the Tyranni examined, and 34 taxa of the Tyranni (including the fossil). The character matrix (Supplementary Table 5), is derived from the characters observed

(Supplementary Tables 1, 2). *Gerygone flavolateralis* (Passeri, Acanthizidae) and *Acanthisitta chloris* (Acanthisittidae) were chosen as respective outgroup taxa to the Tyranni.

The taxon-character matrix was analysed with parsimony using PAUP*4.a166. The executable data matrix with PAUP commands is appended as Nexus File (in Supplementary information). Both parsimony analyses were performed with characters ordered. Gaps were treated as missing data. Parsimony analyses treated all changes as equal ("unweighted") and used heuristic searches with tree-bisection-reconnection (TBR) branch swapping and other default settings, and 1000 random addition replicates per search. Strict consensus trees were computed from the set of most parsimonious trees, and clade support was assessed by bootstrapping using the same settings and 1000 replicates.

Osteological characters used in the phylogenetic analysis.

Feather crest: 0, absent; 1, present but different of fossil; 2, present and similar of fossil.

Relative size of orbits: 0, more than half of the skull; 1, less than half of the skull.

Shape of orbits: 0, the dorsal part is flatter, 1, rounded; 2, almost circular.

Size of nasal opening: 0, wide opening; 1, intermediate size, 2, small opening.

Fenestra antorbitalis (proportion): the proportional size of the fenestra antorbitalis compared to that of the skull. 0, developed; 1, reduced.

Os lacrimale: 0, free; 1, present and more or less individualized.

Latero-dorsal part of ectethmoid: 0, little developed; 1, developed; 2, well developed; 3, highly developed.

Lateral parts of etecthmoid: 0, reduced gap; 1, intermediate gap; 2, large gap.

Crest/ridge on culmen: 0, absent; 1, present.

Foramen (foramina) of coracoid omal end: 0, present, 1, absent.

Processus procoracoideus: 0, little developed; 1, well developed.

Processus acrocoracoideus: 0, little developed; 1, well developed.

Brachial tuberosity (tuberculum brachiale): 0, little developed; 1, developed; 2, well developed.

Sulcus medialis supracoracoidei: 0, straight; 1, rounded; 2, almost circular.

Fossa pneumotricipitalis: 0, single fossa; 1, double fossa: present of an additional fossa.

Crista deltopectoralis: 0, developed; 1, reduced.

Crus ventralis fossae: 0, little developed; 1, well developed.

Crus dorsalis fossae: 0, little developed; 1, well developed.

Shaft shape: 0, curved; 1, not curved.

Depth of incisura capitis: 0, shallow; 1, moderately deep; 2, deep.

Processus flexorius: 0, dorso-caudally deflected edge; 1, straight edge.

Processus supracondylaris dorsalis: 0, double; 1, single.

Processus supracondylaris dorsalis: 0, little developed; 1, developed; 2, well developed.

Tuberculum ligamenti collateralis ventralis: 0, projecting; 1, not projecting.

Cotyla ventralis: 0, ventral edge developed and almost circular; 1, convex ventral edge.

Cotyla dorsalis: 0, little developed; 1, large.

Position of the dorso-proximal edge of the incisura tendinosa (for mm extensor metacarpi ulnaris and extensor digitorum communis), distal ulna: 0, proximal; 1, distal.

Papillae remigales caudales: 0, almost absent; 1, little developed; 2, developed; 3, well developed.

Processus dentiformis: 0, absent; 1, little developed: edge with a slightly wavy; 2, developed; 3, well developed.

Position of processus intermetacarpalis: 0, proximal; 1, more proximal.

Distal symphysis of os metacarpale minus: 0, the os metacarpale minus is lightly protruding, and forms a marked square projection; 1, the distal end of os metacrpale minus is moderately

protruding and its cranial portions forms a projection that reaches farther distally than the facies rticularis digitalis minor (Mourer-Chauviré et al., 1989; Mayr and Manegold 2006).

Processus extensorius: 0, not ventrally deflected; 1, ventrally deflected; 2, slightly ventrally dejected.

Blade of Phx 1 digiti majoris: 0, straight edge; 1, slightly rounded edge; 2, rounded edge (Manegold, 2005).

Processus internus indicis: 0, present ; 1, absent.

Femur: hollow just distal to proximal articular surface, caudal side: 0, absent; 1, shallow; 2, marked.

Medial crest (tibiotarsis proximal most shaft): 0, absent; 1, present.

Tiny proximo-distal groove between the tuberositas retinaculi extensorius lateralis and the tuberculum retinaculi m fibularis, lateral side of distal end, proximal to the lateral condyle, rostral aspect: 0, absent; 1, it is located relatively distally; 2, it is located more proximally.

Fusion (continuity) between crista med plantaris and crista med hypotarsi: 0, absent; 1, present.

Ossified pons supratendineus: 0, not ossified, 1, ossified.

Position of pons supratendineus: 0, not ossified, 1, located proximally; 2, located proximally; 3, located less proximally; 4, located in an intermediate position.

Trochlea metatarsi II: 0, as distal as trochlea metatarsi III; 1, shorter than trochlea metatarsi III; 2, longer than trochlea metatarsi III.

Trochlea metatarsi IV: 0, as distal as trochlea metatarsi III; 1, shorter than trochlea metatarsi III; 2, longer than trochlea metatarsi III.

Supplementary Figures

Supplementary Fig. 1 Strict consensus tree, hardly resolved. As for the bootstrap analysis (Supplementary Fig. 2), the poor resolution and support convey pervasive homoplasy. The fossil is associated in a clade with five members of the Pipridae, although five other extant piprids are more basally misplaced in a wide unresolved polytomy with many Tyrannides. The fossil is, as in the bootstrap analysis, distant from *Sapayoa aenigma*, which is correctly placed within the Eurylaimides

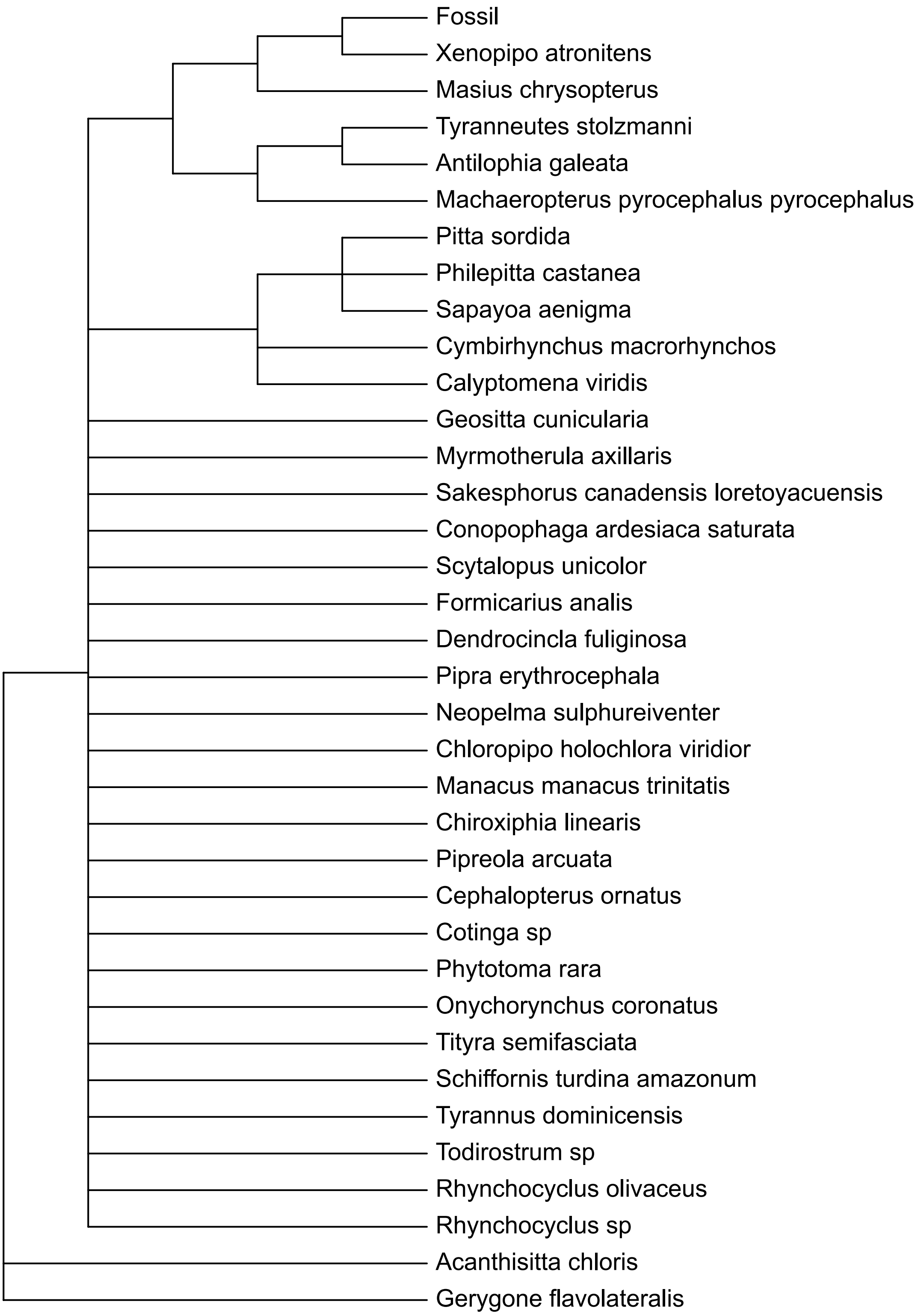
Fossil
Xenopipo atronitens
Masius chrysopterus
Tyranneutes stolzmanni
Antilophia galeata
Machaeropterus pyrocephalus pyrocephalus
Pitta sordida
Philepitta castanea
Sapayoa aenigma
Cymbirhynchus macrorhynchos
Calyptomena viridis
Geositta cunicularia
Myrmotherula axillaris
Sakesphorus canadensis loretoyacuensis
Conopophaga ardesiaca saturata
Scytalopus unicolor
Formicarius analis
Dendrocincla fuliginosa
Pipra erythrocephala
Neopelma sulphureiventer
Chloropipo holochlora viridior
Manacus manacus trinitatis
Chiroxiphia linearis
Pipreola arcuata
Cephalopterus ornatus
Cotinga sp
Phytotoma rara
Onychorynchus coronatus
Tityra semifasciata
Schiffornis turdina amazonum
Tyrannus dominicensis
Todirostrum sp
Rhynchocyclus olivaceus
Rhynchocyclus sp
Acanthisitta chloris
Gerygone flavolateralis

Supplementary Fig. 2 Bootstrap analysis, with 1000 replicates. Only four nodes are supported by the bootstrap, but with a very low value (between 37 and 40%). As for the strict consensus (Supplementary Fig. 1), the poor resolution and support convey pervasive homoplasy. The fossil is however, as in the strict consensus, distant from *Sapayoa aenigma*, which is correctly placed within the Eurylaimides

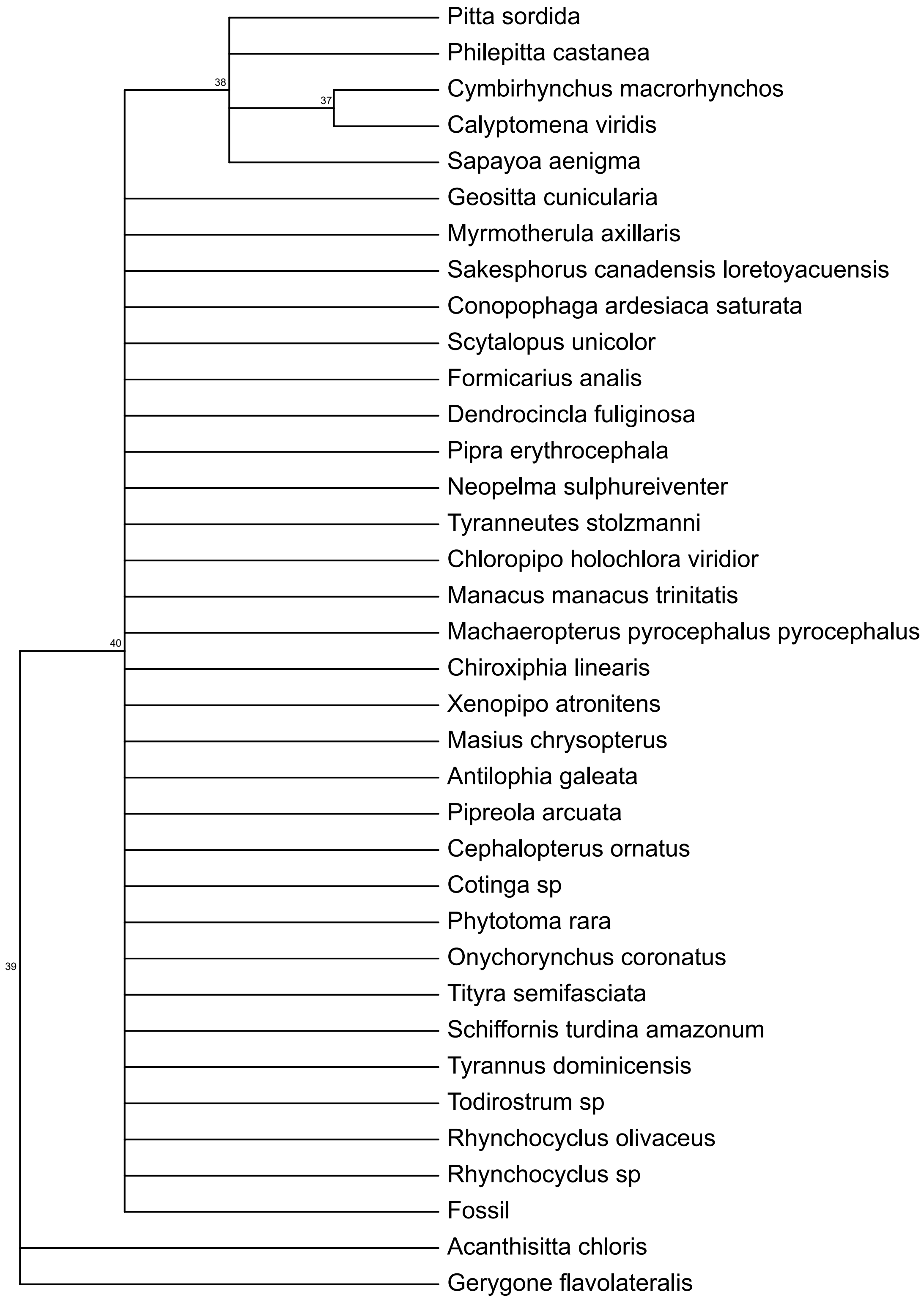

Pitta sordida
Philepitta castanea
Cymbirhynchus macrorhynchos
Calyptomena viridis
Sapayoa aenigma
Geositta cunicularia
Myrmotherula axillaris
Sakesphorus canadensis loretoyacuensis
Conopophaga ardesiaca saturata
Scytalopus unicolor
Formicarius analis
Dendrocincla fuliginosa
Pipra erythrocephala
Neopelma sulphureiventer
Tyranneutes stolzmanni
Chloropipo holochlora viridior
Manacus manacus trinitatis
Machaeropterus pyrocephalus pyrocephalus
Chiroxiphia linearis
Xenopipo atronitens
Masius chrysopterus
Antilophia galeata
Pipreola arcuata
Cephalopterus ornatus
Cotinga sp
Phytotoma rara
Onychorynchus coronatus
Tityra semifasciata
Schiffornis turdina amazonum
Tyrannus dominicensis
Todirostrum sp
Rhynchocyclus olivaceus
Rhynchocyclus sp
Fossil
Acanthisitta chloris
Gerygone flavolateralis
38
37
40
39

Supplementary Fig. 3 Distal part of the tarsometatarsus and toe bones (both right side, in dorsal view) in (A) *Antilophia galeata* (Pipridae) and (B) the fossil NT-LBR-014. There is no fusion at the level of pedal phalanges bones, despite *A. galeata* exhibiting syndactyly of toes III and IV in life, and this is because syndactyly affects only the soft tissues surrounding the bones. I, first digit, positioned in distal extension in (A), in proximal extension in (B); II, second digit –only the first phalanx was preserved attached in the preparation of specimen (A); III, third digit; IV, fourth digit –only the first two phalanges were preserved attached in the preparation of specimen (A); mtI, first metatarsal; tmt, tarsometatarsus. In the fossil (B) the first phalanx of digit II presents proximally a sort of bulb that seems to be pathological. Scale bar, 1 cm

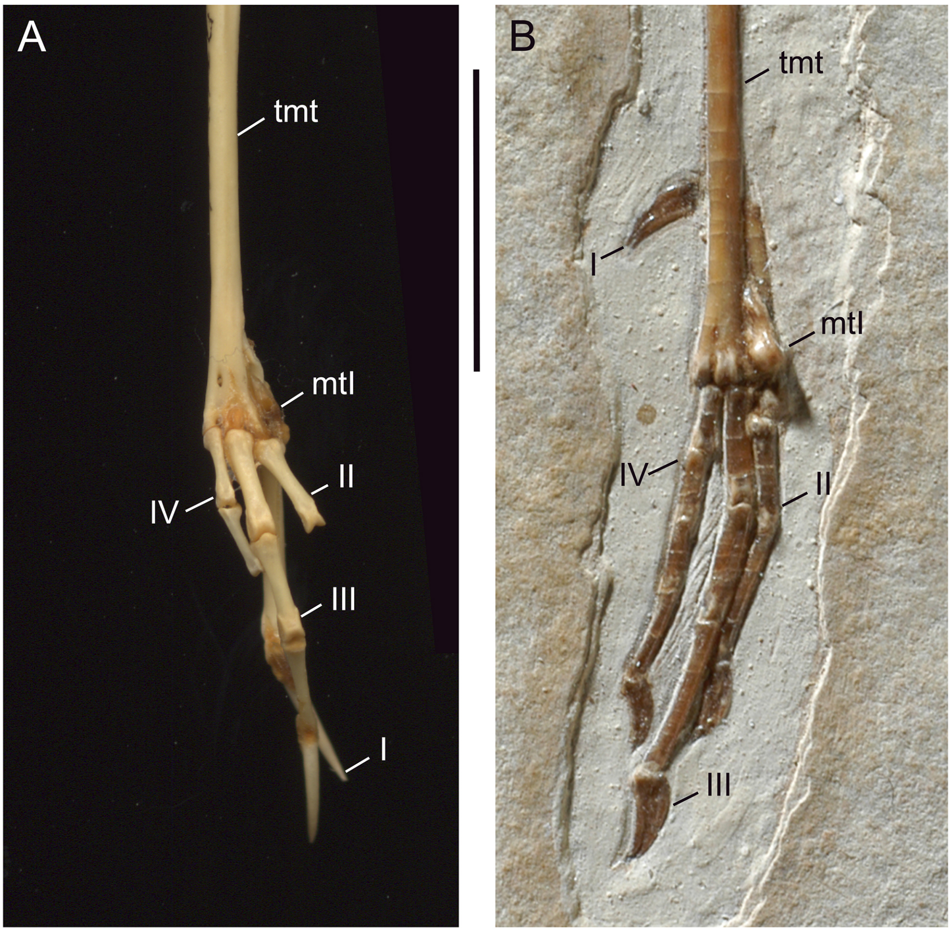

Supplementary Fig. 4 Simpson's diagram of intersegment proportions of the Luberon fossil NT-LBR-014, compared with extant species of the Tyranni. The differences in Log10 of length of the main elements are expressed for the fossil and ten species of Tyranni

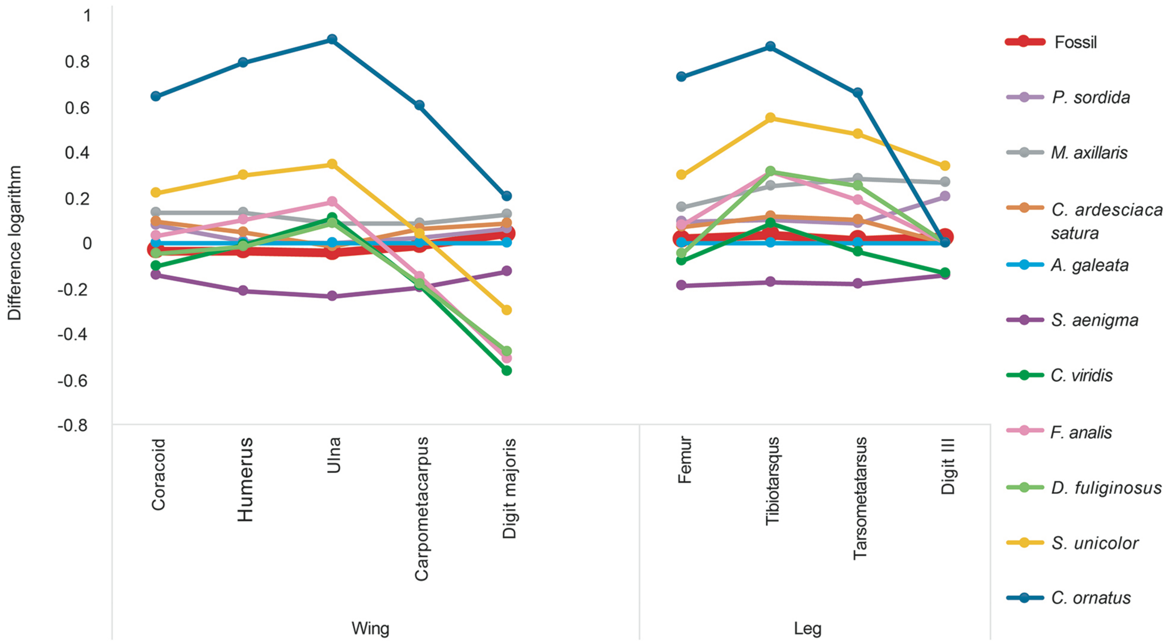

Supplementary Tables

Supplementary Table 1 Osteological characters as observed in the fossil passerine NT-LBR-014, and extant representatives of Tyranni families, Acanthisittidae, basal and other Passeri, and *Wieslochia weissi*, passerine from the Oligocene of Germany.

All character states of species compared to the fossil are given relative to state of the fossil. Only characters that discriminate at least one species among the Tyranni and Acanthisittidae examined have been selected here. *On occasions, character states are given not only for the extant species examined here for comparison, but for more species in the family (in such instances references are given via number in parentheses). (1) it is mentioned if other extant taxa in every genus or family concerned exhibit a crest approaching the fossil's (after del

Hoyo et al., 2019[1]). (2) from this work, and characters 22 and 55 in James et al., 2003[2]. (3) from this work and Mourer-Chauviré et al., 1989[3]; NB: C. Mourer-Chauviré (pers. comm.) noticed (in Smithsonian Institution collections) that among 21 extant species of Furnariidae, only one exhibited a slight undulation, the others none (straight edge of major metacarpal), and among 30 species of Formicariidae, only four possessed a proc. dentiformis (the others nothing: straight edge of major metacarpal). (4) from Boles, 2006[4]. (5) from this work, Millener, 1988[5], and Millener and Worthy, 1991[6]. (6) sensu del Hoyo et al., 2019[1], i.e. comprising taxa earlier in the former Dendrocolaptidae. (7) also from Fjeldsa et al., 2005[7]. (8) a few genera and species exhibit a crest that is different from that of NT-LBR-014, triangular, often acute, pointing upwards over the head, and spread caudally (some *Leptasthenura* spp., *Furnarius cristatus*, *Coryphistera alaudina*, and more or less in the different species of *Pseudoseisura*). (9) same in *Pseudoseisura*[8]. (10) a few genera and species exhibit a crest that is different from that of NT-LBR-014, triangular, often acute, pointing dorso-caudally over the head, and spread caudally. *Pithys albifrons* exhibits original long, upright white tufts on both sides of forehead, shorter tufts below chin. (11) from this work and Feduccia and Olson, 1982[9]. (12) In *Rhinocrypta* a crest extends from the back of the head, that can be spread upwards, making an acute triangle. In *Merulaxis* feathers above the base of rostrum make a « crest » resembling that of *Calyptomena* but sparser. (13) An ossified bridge exists in *Melanopareia* (recently placed in own family[1])[9]. (14) Quadrate even more different in *Melanopareia*[9]. (15) Apart from *Masius, Antilophia* and *Chiroxiphia* (see Table), feather ornaments over the head in the Pipridae exist also in *Ilicura militaris* (where it consists of short, round feather fluffy crest over rostrum base ; more developed in male). (16) Apart from *Cephalopterus* and *Rupicola* (see Table), in the Cotingidae only *Phytotoma* exhibits feather head ornament, especially *P. raimondii* (rear head, spreading upward). (17) In the Tyrannidae, in addition to some species of *Contopus* (short, slight crest back to top of

head ; see Table), crests exist in several genera and species, where always at the back of head, forming when spread a crest with longest feathers toward the top of head (extent of crest variable among genera and species concerned). (For instance also the crest of *Comptostoma* is more on top of head and shorter (and still toward back)). An exception consists in *Anairetes* (variable among species) of a prominent frontal crest of long feathers implanted over the rostrum base, and pointing upwards and backwards ; they form either a fan or a bifurcated crest (like horns) of long, thin feathers. (18) from this work, Feduccia and Olson, 1982[9] and Rich et al., 1985[10]. (19) from Feduccia and Olson, 1982[9], and Rich et al., 1985[10]. (20) data also from Bock, 1963[11] and Olson et al., 1983[12] (NB: *Turnagra* now known to be in the Oriolidae[13]). (21) data also from Bock, 1963[11]. (22) The fossa is simple in all the Passeriformes except most of the the Oscines Passerida - and a few Oscines Corvoidea - where derived states of a double fossa occur polyphyletically; pneumatic in Corvoidea, either pneumatic or non-pneumatic in Passerida[2,3,14]. (23) A processus dentiformis is present in most of the Passerida, while within the Corvoidea it is either absent or present[3,15,16]. (24) Oligocene, Wiesloch-Frauenweiler (Germany); assigned to stem Passeriformes or stem Tyranni[17,18]. NB: for several modern specimens, character states are absent due to the lack of the element or element part, or the incomplete specimen preparation, obscuring detail by ligaments for instance. It is specified for which specimens the skull only could be examined.

Taxon				Skull												Coracoid					
Suborder to Family			Species (species seen for comparison are listed, but see *)	Cranium shape (lateral profile)	Feather crest (1)	Relative size of orbits	Shape of orbits	Osseous nasal septum	Fenestra antorbitalis (proportion)	Os lacrimale	latero-dorsal part of ectethmoid	Interorbital fenestrae (2)	Quadrate shape	Crest/ridge on culmen	Rostrum and mandible (mdb)	Foramen (foramina) of coracoid omal end	Processus procoracoideus	Processus acrocoracoideus	Brachial tuberosity (tuberculum brachiale)	Sulcus medialis supracoracoidei	Coracoid blade shape
Revest-des-Brousses fossil (early Oligocene) NT-LBR-014				see illustrations	over base of rostrum: crest 'triangular', directed forward, with tip recurved slightly backwards	large (11.5mm: more than half of the cranium)	much rounded	present over most of nasal aperture (rostrally)	reduced; equilateral triangle	present and individualized	well developed	dorsal smaller than ventral ; separation rather narrow	see illustrations	no	relatively straight; nasal opening 2/3 of rostrum; mdb with marked gonys	yes	developed	developed	very developed; straight	relatively circular edge	see illustrations
Acanthisittidae			*Acanthisitta chloris* (5)	variable	none	(generally more reduced)	rounded	none	larger (variable)	free ?	very slightly developed	dorsal fenestra proportionally larger ; separation very thin	processus orbitalis much reduced and obtuse; proc. oticus wider	no	beak on average longer and thinner	no	poorly developed, turned 90°	not developed medially	?	?	similar
Tyranni (Suboscines)	Eurylaimides	Pittidae	*Pitta sordida*	similar	none	similar	dorsal edge flatter	present over most of nasal aperture except ventral edge	similar	free ?	developed	ventral fenestra slightly larger	rather similar	no	nasal opening only 1/2of rostrum ; rostrum straighter; mdb without gonys	yes	developed, pointed distally	very slightly less developed medially	less developed	relatively straight edge	processus lateralis more obtuse
		Philepittidae	*Philepitta castanea*	forehead flatter	none	similar	dorsal edge flatter	present over dorsal and rostral large border of the nasal aperture	similar	free ?	slightly developed	dorsal fenestra larger, and separation thinner	slightly different	no	beak thinner, longer, decurved and pointed	yes	developed, but elongation more proximal than in the fossil	less developed medially	?	?	similar
		Eurylaimidae	*Cymbirhynchus macrorhynchos*	rather similar (forehead slightly flatter)	none	similar	dorsal edge flatter	?	more extended dorsally, shape more elongated and narrow	free ?	moderately developed	both fenestrae much larger, separation much thinner	processus orbitalis shorter, processi mandibularis and oticus more curved	no	beak more curved, hooked and robust	yes; smaller but deep	not much developed	less developed medially, more hooked	much less developed	straight edge	similar
		Calyptomenidae	*Calyptomena viridis*	rather similar (forehead slightly flatter)	more reduced, rounded, directed forward	similar	dorsal edge flatter	present only over dorsal and rostral border of the nasal aperture	more extended dorsally, more rounded	free ?	moderately developed	ventral fenestra slightly reduced, dorsal slightly larger	different	yes, small	beak more curved and hooked	yes	developed, same shape as fossil; shorter	less developed medially	far less developed	relatively straight edge	processus lateralis more developed laterally
		Sapayoidae	*Sapayoa aenigma*	identical	none	identical	dorsal edge flatter	almost complete (starting from dorsal edge)	similar	free ?	slightly developed	ventral fenestra reduced, separation slightly thinner	similar	no	rostrum similar; mdb without gonys	yes	developed, same shape as fossil	very slightly less developed medially and pointed	?	?	rather similar
	Tyrannides	Furnariidae (6)	*Geositta cunicularia*	rather similar (7)	none (8)	similar (7)	dorsal edge slightly flatter	none / oblique bar	generally wider	absent or fused	moderately developed	rather similar to fossil ; fenestrae can be fused (no separation)	very similar	no	beak thinner, longer, decurved and pointed; nasal opening reduced; mdb gonys less marked (1)	yes	moderately developed, pointed rather acutely	slightly less developed medially	not much developed	more rounded edge	processus lateralis more extended latero-distally
			Dendrocincla fuliginosa	forehead slightly flatter	none	similar	rounded	none	larger	absent or fused	well developed	ventral fenestra much smaller	proc. orbitalis narrower	no	beak much longer; nasal opening reduced	yes	developed, thinner, and shape different	very slightly less developed medially	developed	rounded edge	processus lateralis much more extended latero-distally
		Thamnophilidae	*Myrmotherula axillaris*	rather similar	none (10)	smaller	dorsal edge slightly less rounded	almost complete (except rostralmost part)	slightly larger	absent or fused	developed	dorsal fenestra larger and separation much thinner	proc. orbitalis narrower	no	beak thinner, straighter; no gonys	?	moderately developed, pointed rather acutely	slightly less developed and pointed medially	developed	rounded edge	processus lateralis more obtuse
			Sakesphorus canadensis	rather similar (forehead slightly flatter)	crest on the back of head (can be spread upwards)	similar	rounded	complete	slightly larger	absent or fused	well developed	dorsal fenestra slightly larger, and separation thinner	proc. orbitalis proportionally longer	no	beak longer; nasal opening reduced; gonys slightly reduced	?	slightly less developed	point slightly less shifted medially	?	?	processus lateralis developed slightly more distally
			Thamnophilus doliatus (skull)	forehead flatter	present; directed dorsally	smaller	rounded	almost complete	larger, more rounded	absent or fused	developed	similar to fossil ; separation thin	proc. orbitalis narrower	no	beak longer and more curved	?	?	?	?	?	?
			Thamnophilus coecus (skull)	forehead flatter	none	similar	much rounded	almost complete	more rounded	absent or fused	very slightly developed	no dorsal fenestra	?	no	beak longer and more hooked	?	?	?	?	?	?
		Conopophagidae	*Conopophaga ardesiaca saturata*	similar	none	similar	rounded	over the caudal 3/4	slightly larger	absent or fused	slightly developed	dorsal fenestra larger, separation thinner	proc. orbitalis narrower	no	beak longer beyond nasal opening	?	not much / moderately developed; obtuse corner shaped end	slightly less developed medially	much less developed	very rounded edge	similar
		Rhinocryptidae	*Scytalopus unicolor*	slightly flatter	none (12)	smaller (11)	dorsal edge flatter	none / narrow oblique bar	variable	absent or fused	very slightly - to developed	single large fenestra (no ossified septum/separation) (13)	rather similar (14)	yes / no (11)	rostrum thinner and more hooked; mdb thinner, decurved, and gonys almost absent	no; (and NB: triosseal canal closed, by junction between processi procoracoideus and acrocoracoideus)	very little developed	not much developed medially	much less developed	very rounded edge	less developed
		Formicariidae	*Formicarius analis*	forehead more angular	none	very slightly smaller	rounded	none	larger	absent or fused	developed	ventral fenestra more developed, separation thinner	similar	no	beak longer; nasal opening reduced; gonys reduced	yes	not much / moderately developed; acute corner shaped end	less developed medially	much less developed	very rounded edge	processus lateralis developed more distally
		Pipridae	*Ceratopipra erythrocephala*	similar	none (15)	similar	much rounded	over rostral and dorsal border	larger and slightly more rounded	present and more or less individualized	developed	dorsal fenestra more developed, ventral fenestra more reduced, separation thinner	very similar	no	similar to fossil; gonys reduced	yes	developed, slightly more external	developed	?	?	?
			Neopelma sulphureiventer	rather similar (forehead slightly flatter)	none	slightly smaller	rounded	narrow over dorsal edge	much larger, more rounded, and more developed rostrally	?	developed	dorsal fenestra more developed ; ventral fenestra more reduced	more robust; processus orbitalis less developed	no	similar	yes; smaller	developed; straighter and more massive	developed; more hooked; thinner	slightly less developed	rounded edge	processus lateralis more developed distally
			Tyranneutes stolzmanni	similar	none	similar	much rounded	narrow over dorsal edge	slightly larger	?	developed	dorsal fenestra more developed ; ventral fenestra more reduced	proc. orbitalis more ventral	no	beak thinner; nasal opening reduced; gonys reduced	yes	developed, slightly more external	slightly less shifted medially	slightly less developed	very rounded edge	similar
			Chloropipo holochlora viridior	forehead slightly flatter	none	similar	rounded	narrow over dorsal and rostral edge	similar	?	developed	dorsal fenestra larger, ventral fenestra smaller	?	no	beak straighter, with edges more 'parallel'; gonys reduced	yes	similar, slightly more external	slightly less pointed medially	slightly less developed	rounded edge	rather similar
			Manacus manacus	forehead slightly flatter	none	similar	rounded	over rostral half, and also a narrow central caudal extension	slightly larger	?	moderately developed	dorsal fenestra larger, ventral fenestra smaller	proc. orbitalis more ventral	no	beak more decurved; gonys slightly reduced	?	more squarish; very slightly more external	slightly less pointed	slightly less developed	very rounded edge	slightly more developed distally
			Machaeropterus pyrocephalus	forehead flatter, cranium flatter	none	similar	rounded	oblique narrow central extension	slightly larger	?	developed	dorsal fenestra larger, ventral fenestra smaller	proc. orbitalis more ventral	no	mdb slightly more decurved with an angle	?	wider and more obtuse; square	point les shifted medially	much less developed	very rounded edge	similar
			Chiroxiphia linearis	forehead slightly flatter	short, round feather fluffy crest over rostrum base	similar	rounded	over 4/5 rostro-dorsal part, with narrow caudo-ventral extension	slightly larger	suture visible	developed, round and obtuse	dorsal fenestra larger, ventral fenestra smaller	proc. orbitalis slightly more ventral	no	beak smaller, shorter and much more curved; nasal opening reduced	?	more tapering	slightly less pointed	slightly less developed	rounded edge	similar
			Xenopipo atronitens	forehead slightly flatter	none	similar	rounded	over dorsal half	identical	present, individualized, fusion with suture visible	well developed, similar to fossil	ventral fenestra smaller, dorsal fenestra larger ; separation slightly thinner	proc. orbitalis slightly more ventral	no	beak shorter, decurved; gonys absent	yes	similar, slightly more external	developed slightly less medially	slightly less developed; hooked	relatively circular edge	more developed laterally
			Masius chrysopterus	forehead slightly concave	crest over rostrum base: ponted, directed forward and recurved forward	similar	rounded	over dorso-caudal half	(shape similar but) slightly larger	present and more or less individualized	well developed, similar to fossil	ventral fenestra smaller, dorsal fenestra larger ; separation slightly thinner	proc. orbitalis slightly more ventral	no	beak shorter; mdb much more decurved, and gonys slightly reduced	yes	only slightly wider	less developed medially	slightly less developed; hooked	relatively circular edge	similar
			Antilophia galeata	similar	crest over rostrum base **identical** to fossil's	similar	rounded	over more than 3/4 dorsal	slightly larger	present and more or less individualized	developed	dorsal fenestra larger, ventral fenestra smaller	similar	no	similar; but gonys less marked	yes	as fossil	developed; shape similar	similarly very developed; slightly more hooked	very rounded edge	similar
		Cotingidae	*Pipreola arcuata*	forehead flatter ; cranium more elongated	none	similar	rounded	over 1/3 rostral	shape similar but larger	present and more or less individualized	well developed	dorsal fenestra much larger, ventral fenestra smaller	more robust; processus orbitalis less developed	no	beak more hooked and robust; mdb more curved; gonys less developed	yes: fenestra	developed; more developed anteriorly	slightly less developed	slightly less developed	very rounded edge	more developed distally
			Cephalopterus ornatus	flatter	immense crest over head, recurved forward and reaching level of beak tip	similar	rounded	over 1/3 rostro-dorsal	more extended dorsally	present and more or less individualized	developed	both fenestrae much smaller ; separation wider	proc. orbitalis wider and more ventral	no	beak more developed rostralmost; more hooked; gonys almost absent	yes	not much developed	developed	similarly very developed	relatively rounded edge	much more developed distally
			Cotinga sp	flatter	none (16)	similar	dorsal edge slightly flatter	over more than 3/4 caudal	rather similar	present and more or less individualized	well developed	both fenestrae much smaller, (dorsal fenestra larger than the ventral)	proc. orbitalis more ventral	no	rostrum slightly longer and more hooked; mdb much thinner and straighter, without gonys	yes	slightly less developed	slightly less pointed medially	similarly very developed	relatively rounded edge	similar
			Procnias sp. (skull)	similar	none	similar	rounded	thin ; almost complete ventrally	more comma-shaped, pointed ventro-caudally	present and more or less individualized	well developed	One single large fenestra	?	no	beak shorter; nasal opening smaller	?	?	?	?	?	?
			Pipreola intermedia signata (skull)	forehead flatter	none	similar	rounded	thin ; almost complete ventrally	similar	present and fused	well developed	One single large fenestra, no ossified septum	?	no	mdb slightly more curved	?	?	?	?	?	?
			Rupicola rupicola	forehead flatter	present ; fan-shaped	similar	rounded	almost completely closing the nasal aperture, viewed in lateral aspect (except thin ventral limit)	more rounded, and comma-shaped, pointed ventro-caudally	present and more or less individualized	slightly developed	One single large fenestra, no ossified septum	?	no	beak more hooked; mdb thinner	yes	very little developed	much less developed	slightly less developed	rounded edge	rather similar
			Phytotoma rara	forehead flatter	none (16)	similar	rounded	almost complete dorsally	larger, more rectangular	present and more or less individualized	developed	dorsal fenestra larger, ventral fenestra much reduced, and round	processus orbitalis much narrower, processus mandibularis wider	no	beak more hooked, nasal opening more reduced, absence of gonys	yes	less developed	slightly less developed	slightly less developed	relatively rounded edge	processus lateralis more developed medially
		Tityridae	*Oxyruncus cristatus*	forehead flatter ; cranium more elongated	none	similar	rounded	dorsal, thin septum more developed (high) rostrally	similar	present and more or less individualized	developed	dorsal fenestra much reduced ; ventral fenestra even more reduced	?	no	beak thinner	yes	developed, as fossil	less developed; smaller	similarly very developed; more hooked	rounded edge	processus lateralis thinner and more curved
			Onychorhynchus coronatus	forehead flatter ; cranium more elongated	large, semi-circular crest, fan-shaped ; nuchally ; same for other species in the genus	similar	rounded	over 1/3 ventral	larger, more developed dorsally	present and more or less individualized	developed	both fenestrae much smaller	processus mandibularis more developed laterally	no	beak longer rostralmost, and more hooked; nasal opening more reduced; gonys very slight	yes	slightly less developed, thinner than fossil	much less developed	slightly less developed	rounded edge	processus lateralis more developed laterally and more curved
			Tityra semifasciata	forehead flatter	none	similar	rounded	semi-circular small rostro-ventral open area	larger	free?	well developed	both fenestrae larger	processus orbitalis much more developed rostrally; processus mandibularis more developed laterally	no	nasal opening reduced, absence of gonys	yes	much thinner	less developed	slightly less developed	relatively straight edge	processus lateralis more developed medially and laterally
			Schiffornis turdina	similar	none	similar	rounded	narrow oblique central bar, directed from rostro-dorsal to caudo-ventral	larger	well individualized; reaches the ectethmoid	well developed	ventral fenestra slightly reduced ; dorsal fenestra larger	proc. orbitalis more ventral	no	similar; but gonys less marked and beak slightly decurved	yes: fenestra	wider and more othogonal	less pointed	slightly less developed	rounded edge	more developed latero-distally
		Tyrannidae	*Tyrannus dominicensis*	rather similar	none (17)	rather similar	?	over 1/3 dorsal	?	present and more or less individualized	developed	ventral fenestra much reduced ; dorsal fenestra larger	?	no	beak longer; gonys less marked	yes	developed, same shape as fossil	less developed medially	much less developed	relatively straight edge	rather similar
			Tyrannus sp.	forehead much flatter	slight crest in dorsal position	similar	dorsal edge flatter	over rostro-dorsal half	larger	?	slightly developed, pointed	ventral fenestra larger, separation thinner	proc. orbitalis shorter and proc. oticus wider	?	beak much longer; gonys less marked	yes	markedly less developed; point in proximal position	less developed medially	slightly less developed; hooked	relatively straight edge	developed more latero-distally
			Todirostrum sp.	similar	none	similar	rounded	over rostro-dorsal half ; very thin	more extended dorsally	free?	slightly developed, pointed	ventral fenestra larger, separation thinner	?	no	beak thinner and more decurved; nasal opening shorter; gonys less marked	yes	markedly less developed	less developed medially	slightly less developed	rounded edge	processus lateralis more developed laterally; more curved
			Contopus latirostris (skull)	forehead much flatter	slight crest in top-dorsal position (short in this species)	similar	rounded	(over large part)	rather similar	?	moderately developed	?	?	?	beak much longer, with edges parallel	?	?	?	?	?	?
			Rhynchocyclus olivaceus	similar	none (17)	similar	rounded	dorsal half and 1/4 rostral	larger	free?	more developed	ventral fenestra more reduced, dorsal fenestra larger	processus mandibularis more shifted laterally	no	beak slightly thinner; slightly more decurved; nasal opening more reduced; gonys less marked	yes	less developed than in the fossil; with an individualized losangic or elliptical flat, oblique area at the extremity, ventral side.	less developed	similarly very developed; slightly hooked	relatively circular edge	more developed medially and laterally
			Rhynchocyclus sp.	similar	slight crest in dorsal position	similar	rounded	dorsal half and 1/4 rostral	larger	free?	more developed	ventral fenestra more reduced, dorsal fenestra larger	?	no	beak slightly thinner; slightly more decurved; nasal opening reduced; gonys less marked	yes	even more developed mesially than fossil; with an individualized losangic or elliptical flat, oblique area at the extremity, ventral side.	less developed	slightly less developed	rounded edge	more developed medially and laterally
Passeri (Oscines)		Menuridae (18)	*Menura novaehollandiae*	?	none	?	?	none	?	present and individualized (free but still in place)	developed	dorsal fenestra much reduced ; separation rather wide	proc. oticus reduced; processus orbitalis reduced and more robust	no	?	yes	undeveloped	?	?	?	?

	Atrichornithidae (19)		?	none	reduced		none	?	present, free and small	(developed)	One single large fenestra, no ossified septum	processus orbitalis reduced and more robust	yes	?	?	very little developed	?	?	?	?
	Ptilonorhynchidae (11)	*Sericulus* sp., *Ptilonorhynchus violaceus*	?	none	large (20)	?	none		present and individualized, large	?	ventral and dorsal fenestrae reduced	proc. oticus proportionally shorter	no	?	yes	obtuse to slightly acute	?	?	?	?
	Climacteridae (2)		?	none	?	?	?	?	free lacrimal absent (2)	?	One single large fenestra, no ossified septum (2)	?	?	?	?	?	?	?	?	?
	Meliphagidae	*Philemon corniculatus, Meliphaga lewini, Manorina melanocephala*	?	none	large	?	none	?	free lacrimal absent (2)	?	ventral and dorsal fenestrae reduced; a third ventral even smaller (2)	proc.orbitalis developed more dorsally	no	?	?	very little developed	?	?	?	?
	Pardalotidae	*Pardalotus punctatus*	?	none	large	?	none	?	?	?	One single large fenestra, no ossified septum	?	no	?	?	undeveloped	?	?	?	?
	Acanthizidae	*Gerygone flavolateralis*	forehead flatter and longer	none	large	dorsal edge flatter	almost complete	larger	fused or absent	slightly developed	dorsal fenestra more developed, ventral fenestra more reduced, separation thinner	?	no	rostrum longer and thinner; mdb gonys less marked	no	less developed	much less developed	similarly very developed; much more hooked	rounded edge	more developed latero-distally
	Pomatostomidae	*Pomatostomus temporalis trivirgatus*	?	none	?	?	?	?	?	?	?	?	?	?	?	very little developed	?	?	?	?
	Paradisaeidae	*Ptiloris paradiseus, Paradisaea minor*	?	present; directed dorsally	large (21)	?	complete	?	present	?	One single large fenestra, no ossified septum	processus orbitalis shorter, slightly narrower, tip expanded	no	?	?	very little developed	?	?	?	?
	Other oscines								many Corvoidea: free lacrimal present; most Passerida: free lacrimal absent (2)	Corvidae: very much developed	many Corvoidea: dorsal fenestra absent; many Passerida: dorsal fenestra present (2)	Corvidae: proc. oticus proportionally shorter	no	?	Corvidae: yes	absent or very little developed (in Corvidae: absent)	Corvidae: developed	?	?	?
Wieslochia weissi † (24)			?	?	more reduced (less than half of the cranium length)	?	absent	?	?	?	?	?	no	?	no	developed	not much developed	?	?	much more developed laterally

Taxon				Scapula	Humerus						Ulna			Carpometacarpus					Proximal wing phalanx of major digit	
Suborder to Family			Species (species seen for comparison are listed, but see *)	**Outline**	**Fossa pneumotricipitalis**	**Crista deltopectoralis**	**Shaft shape**	**Depth of incisura capitis**	**Processus flexorius**	**Processus supracondylaris dorsalis**	**Tuberculum ligamenti collateralis ventralis**	**Cotyla ventralis**	**Papillae remigales caudales**	**Processus dentiformis (3)**	**Processus Intermetacarpalis**	**Distal symphysis of os metacarpale minus**	**Carpometacarpus outline**	**Processus extensorius**	**Blade**	**Processus internus indicis**
Revest-des-Brousses fossil (early Oligocene) NT-LBR-014				see illustrations	unique	see ilustrations	straight	Rather deep	Straight edge	unique, well-marked	prominent	marked groove just distal to the distal border/bulge of cotyla ventr. Plane of cot. ventr. forms an angle with olecranon axis. (ventral view)	rather little marked	present, rather well marked	see illustrations	"suboscine type", ie: short and rounded caudally, and forming a rostrally situated pointed protuberance extending more distally ; NB: pointed protuberance slight	see illustrations	in dorsal aspect, the proximal border of the proc. extensorius forms a right angle with the rostral border of the dorsal edge of the trochlea carpalis, without a notch in the angle.	"suboscine type", ie: caudal edge convex, rounded (with the most caudal extension situated distally)	present
Acanthisittidae			*Acanthisitta chloris* (5)	much more curved distally (except *Dendroscansor*)	Two similar fossae, moderate, non-pneumatic	reduced	curved (S-shaped)	shallow	?	very little marked to absent	prominent	?	marked to very well marked	absent, or marked undulation	more proximal	"suboscine type" without pointed protuberance, to "oscine type" (ie symphysis square shaped)	rather similar	variable between genera of Acanthisittidae (5)	"oscine type", ie: caudal edge straight	reduced to absent
Tyranni (Suboscines)	Eurylaimides	Pittidae	*Pitta sordida*	wider proximally	unique, pneumatic	similar	straight	rather deep	edge deflected dorso-caudally	unique, well-marked	prominent	distal edge of cotyla ventr. marked; no groove distal to it. Plane of cot. ventr. (in ventral view) more in continuity with olecranon axis.	very well marked	present, rather well marked	similar	"suboscine type" with pointed protuberance	slightly less elongated	there is a distinct notch in the angle between the proc. ext. and the dorsal edge of trochlea carpalis.	intermediate between typical suboscine and typical oscine types	present
		Philepittidae	*Philepitta castanea*	rather similar	unique, pneumatic	similar	straight	rather deep	?	unique, well-marked	prominent	?	very well marked	very slight undulation to absent	similar	intermediate between suboscine and oscine types, nearer to suboscine type; with pointed protuberance	slightly less elongated	there is a distinct notch in the angle between the proc. ext. and the dorsal edge of trochlea carpalis.	intermediate between typical suboscine and typical oscine types	present
		Eurylaimidae	*Cymbirhynchus macrorhynchos*	rather similar but wider	unique, pneumatic	similar	straight	moderately deep	edge deflected dorso-caudally	unique, little marked	prominent	distal edge of cotyla ventr. marked; no groove distal to it. Plane of cot. ventr. (in ventral view) more in continuity with olecranon axis.	very well marked	present, rather well marked; situated more proximal	similar but less pointed	suboscine type ; pointed protuberance more developed	slightly more robust	there is a distinct notch in the angle between the proc. ext. and the dorsal edge of trochlea carpalis.	suboscine type, blade edge slightly more rounded	present
		Calyptomenidae	*Calyptomena viridis*	rather similar but more curved distally	unique, pneumatic	similar	straight	rather deep	edge deflected dorso-caudally	unique, little marked	prominent	distal edge of cotyla ventr. marked; no groove distal to it. Plane of cot. ventr. (in ventral view) more in continuity with olecranon axis.	very well marked	absent to rather well marked	similar but less pointed	rather more similar to oscine type (ie square), but with a pointed protuberance	slightly more robust	there is a distinct notch in the angle between the proc. ext. and the dorsal edge of trochlea carpalis.	suboscine type, blade edge slightly more rounded	present
		Sapayoidae	*Sapayoa aenigma*	similar	unique, pneumatic	similar	straight	shallow	edge deflected dorso-caudally	unique, well-marked	prominent	distal edge of cotyla ventr. marked; no groove distal to it. Plane of cot. ventr. (in ventral view) more in continuity with olecranon axis.	moderately better marked	present	more distal	suboscine type; with pointed protuberance	less elongated	there is a distinct notch in the angle between the proc. ext. and the dorsal edge of trochlea carpalis.	similar	present
	Tyrannides	Furnariidae (6)	*Geositta cunicularia*	NB: cranial extremity axially rotated	unique, pneumatic	similar	straight	shallow	straight edge	unique, very little marked	prominent	?	very little marked	absent to very slight undulation	similar	suboscine type; with pointed protuberance	similar	similar	intermediate between typical suboscine and typical oscine types (closer to the latter)	present
			Dendrocincla fuliginosa	rather similar	unique, pneumatic	similar	straight	rather deep	?	unique, very little marked, and an even less marked second one	prominent	?	lightly better marked	absent to very slight undulation	similar	suboscine type	slightly less elongated	similar	rather oscine type	almost absent
		Thamnophilidae	*Myrmotherula axillaris*	more curved and more angular distally	unique, pneumatic	similar	straight	?	?	unique, very little marked	very prominent	?	similar	slight undulation	more square	rather more similar to oscine type (ie square), but with a very slight pointed protuberance	processus extensorius more the shape of an isosceles triangle	?	rather oscine type	absent
			Sakesphorus canadensis	more curved distally	unique, pneumatic	similar	straight	?	?	unique, little marked	prominent	?	similar	absent	rather similar	intermediate between suboscine and oscine types, nearer to suboscine type; with a very slight pointed protuberance	rather similar	?	intermediate between typical suboscine and typical oscine types; rather more like suboscine but narrow	absent
			Thamnophilus doliatus (skull)	?	?	?	?	?	?	?	?	?	?	?	?	?	?	?	?	?
			Thamnophilus coecus (skull)	?	?	?	?	?	?	?	?	?	?	?	?	?	?	?	?	?
		Conopophagidae	*Conopophaga ardesiaca saturata*	much more curved distally	unique, pneumatic	similar	very slightly S-shaped	?	?	unique, well marked, with a very little marked second one	prominent	?	similar	present, very well marked	slightly more developed	rather more similar to oscine type (ie square), but with a pointed protuberance	(slightly more robust)	similar	intermediate between typical suboscine and typical oscine types; rather more like suboscine but narrow	absent
		Rhinocryptidae	*Scytalopus unicolor*	more curved distally	unique, less pneumatic	reduced	S-shaped	rather deep; (all Rhinocryptidae: some shallower (11))	straight edge	unique, well marked	very prominent	?	very little marked	present, very well marked (11)	rather developed	suboscine type	more robust	similar	rather oscine type	almost absent
		Formicariidae	*Formicarius analis*	more curved distally	unique, pneumatic	similar	straight; (all Formicariidae: straight to S-shaped (11))	shallower; (all Formicariidae: some as deep as in fossil (11))	straight edge	unique, rather marked	prominent	?	moderately better marked	very well marked in this species, but in majority absent in this family	slightly more developed	suboscine type; with pointed protuberance	similar	somehow intermediate	intermediate between typical suboscine and typical oscine types (closer to the latter)	very slight
		Pipridae	*Ceratopipra erythrocephala*	slightly more curved	unique, pneumatic	similar	straight	rather deep	straight edge	unique, well marked	prominent	similar	very little marked	absent	similar	suboscine type; with very slight pointed protuberance	similar	similar	suboscine type, very slightly narrower	present
			Neopelma sulphureiventer	?	?	?	?	?	?	?	prominent	similar	unmarked	absent	less developed ; straighter	suboscine type; with slight pointed protuberance	spatium intermetacarpalis less developed distally	similar	?	?
			Tyranneutes stolzmanni	slightly more curved distally	unique, pneumatic	similar	straight	moderately deep	straight edge	unique, well marked	prominent	?	very little marked	absent	similar	suboscine type; with pointed protuberance	similar	similar	suboscine type, but narrower	present
			Chloropipo holochlora viridior	rather similar	unique, pneumatic	similar	straight	shallow	straight edge	unique, well marked	prominent	?	similar	absent	rather similar	suboscine type; with pointed protuberance	rather similar	similar	identical	present
			Manacus manacus	distal two thirds much wider	unique, pneumatic	similar; slightly shorter (prox-distally)	straight	shallow	straight edge	unique, very well marked	prominent	?	similar	present	much more developed	suboscine type; with slight pointed protuberance	more robust	similar	similar	absent
			Machaeropterus pyrocephalus	similar	unique, pneumatic	slightly less developed; slightly shorter (prox-distally)	straight	rather deep	straight edge	unique, well marked	prominent	?	very well marked	slight undulation	slightly more developed	suboscine type; with very slight pointed protuberance	similar	similar	suboscine type, but narrower	present
			Chiroxiphia linearis	similar	unique, pneumatic	similar	straight	rather deep	straight edge	unique, very well marked	prominent	?	similar	absent	similar	suboscine type; with very slight pointed protuberance	similar	similar	suboscine type, but the most caudal extension of the blade is proximal	present
			Xenopipo atronitens	more curved distally	unique, pneumatic	similar	straight	shallow	straight edge	unique, very well marked	prominent	?	very little marked	present	similar	suboscine type; with pointed protuberance	similar	similar	similar	present
			Masius chrysopterus	rather similar	unique, pneumatic	similar	straight	shallow	straight edge	unique, very well marked	prominent	similar	very little marked	very slight	similar	suboscine type; with very slight pointed protuberance	similar	similar	similar	present; reduced
			Antilophia galeata	similar	unique, pneumatic	similar	straight	rather deep	straight edge	unique, well marked	prominent	similar	similar	absent	similar	suboscine type; with very slight pointed protuberance	similar	similar	suboscine type; but narrower	present
		Cotingidae	*Pipreola arcuata*	?	unique, pneumatic	similar	straight	moderately deep	straight edge	unique, well marked	prominent	similar	?	undulation	more pointed	suboscine type; with slight pointed protuberance	shorter, spatium intermetacarpalis less elongated	similar	suboscine type; but narrower	present
			Cephalopterus ornatus	wider proximally	unique, pneumatic	similar	straight	shallow	edge relatively straight	unique, very little marked	prominent	similar	very well marked	absent or slight undulation	more developed	suboscine type; with pointed protuberance	similar	similar	suboscine type ; blade even more prominent caudally	present
			Cotinga sp	area of maximal curvature wider	unique, pneumatic	similar	straight	shallow	straight edge	unique, very little marked, and an even less marked second one	prominent	similar	moderately better marked	undulation	similar	suboscine type; with pointed protuberance	similar	somehow intermediate but closer to fossil	similar	present
			Procnias sp. (skull)	?	?	?	?	?	?	?	?	?	?	?	?	?	?	?	?	?
			Pipreola intermedia signata (skull)	?	?	?	?	?	?	?	?	?	?	?	?	?	?	?	?	?
			Rupicola rupicola	wider; area of maximal curvature more proximal	?	?	?	?	?	?	?	?	?	?	?	?	?	?	?	?
			Phytotoma rara	wider and more curved distally	unique, pneumatic	similar	straight	rather deep	straight edge	unique, very little marked	prominent	similar	very little marked	very slight undulation	more pointed and narrower	suboscine type; with pointed protuberance	similar	?	very similar	present
		Tityridae	*Oxyruncus cristatus*	?	?	?	?	?	?	?	prominent	similar	similar	?	?	?	?	?	?	?
			Onychorhynchus coronatus	wider and more curved distally	unique, pneumatic	similar	straight	rather deep	straight edge	unique, rather marked	prominent	similar	similar	slight undulation	more developed laterally	suboscine type; with pointed protuberance	spatium intermetacarpalis more developed	similar	shape similar; pila cranialis slightly more individualized and protruding in relief ventrally, relative to the blade plane	present
			Tityra semifasciata	wider	unique, non-pneumatic	similar	straight	rather deep	straight edge	unique, well marked	prominent	similar	very little marked	absent	slightly more developed	suboscine type; with pointed protuberance	trochlea carpale more developed	similar; proc. ext. more developed	shape similar; pila cranialis more individualized and protruding in relief ventrally, relative to the blade plane	present
			Schiffornis turdina	more curved distally	unique, pneumatic	similar	straight	rather deep	straight edge	unique, well marked	prominent	?	(very) little marked	slight undulation	similar	suboscine type; with pointed protuberance	similar	similar	very similar	present
		Tyrannidae	*Tyrannus dominicensis*	similar	unique, pneumatic	similar	straight	rather deep	straight edge	unique, rather little marked	prominent	similar	moderately better marked	slight undulation	slightly less developed	suboscine type; with pointed protuberance	similar	similar	very similar	present
			Tyrannus sp.	?	?	?	?	?	?	?	?	?	?	?	?	?	?	?	?	?
			Todirostrum sp.	much wider and more curved	unique, pneumatic	similar	straight	shallow	straight edge	unique, very little marked	prominent	similar	similar	absent	similar	suboscine type; with pointed protuberance	similar	similar	very similar	present
			Contopus latirostris (skull)	?	?	?	?	?	?	?	?	?	?	?	?	?	?	?	?	?
			Rhynchocyclus olivaceus	more curved distally	unique, pneumatic	similar	straight	shallow	straight edge	unique, rather marked, wider	prominent	similar	very little marked	undulation	less pointed medially ; narrower	suboscine type; with pointed protuberance	similar	similar	shape similar; pila cranialis more individualized and protruding in relief ventrally, relative to the blade plane; blade more rounded.	present
			Rhynchocyclus sp.	more curved	unique, pneumatic	similar	straight	shallow	straight edge	almost absent ; and beginning of a second	prominent	similar	almost absent	slight undulation	slightly more pointed medially	suboscine type; with pointed protuberance	similar	similar	shape similar; pila cranialis more individualized and protruding in relief	present

																		ventrally, relative to the blade plane	
Passeri (Oscines)	Menuridae (18)	*Menura novaehollandiae*	curved, more homogeneously	unique, pneumatic	reduced	straight	?	?	unique, little marked	non-prominent: flat	?	moderately marked	present, very well marked	developed	oscine type	more robust	?	?	?
	Atrichornithidae (19)		curved, more homogeneously	unique, pneumatic	reduced	S-shaped	?	?	absent	non-prominent: flat	?	rather marked	slight	?	oscine type	?	?	oscine type (blade caudal edge straight)	?
	Ptilonorhynchidae (11)	*Sericulus* sp., *Ptilonorhynchus violaceus*	curved, more homogeneously	unique, pneumatic	similar	straight	?	?	marked, sometimes beginning of a second	non-prominent: almost flat	?	little marked	absent	similar	oscine type	similar	?	oscine type	absent
	Climacteridae (2)		?	unique, non-pneumatic (2)	?	?	?	?	?	?	?	?	?	?	?	?	?	?	?
	Meliphagidae	*Philemon corniculatus, Meliphaga lewini, Manorina melanocephala*	straighter and wider proximally	unique, pneumatic (2); (beginning of a second sometimes rather marked)	similar	straight	?	?	double, little developed	non-prominent: flat	?	?	extremely slight undulation	?	oscine type	?	?	oscine type	absent
	Pardalotidae	*Pardalotus punctatus*	more curved and more angular distally	unique, pneumatic	similar	straight	?	?	unique, very well marked	?	?	?	?	?	?	?	?	?	?
	Acanthizidae	*Gerygone flavolateralis*	rather similar	double ; dorsal fossa pneumatized	similar	straight	rather deep, very narrow	straight edge	prominent (double but with very shallow division)	non-prominent: flat	distal edge of cotyla ventr. marked; no groove distal to it. Plane of cot. ventr. (in ventral view) more in continuity with olecranon axis.	moderately marked	very well marked	more proximal, less developed	oscine type	similar	similar	?	?
	Pomatostomidae	*Pomatostomus temporalis trivirgatus*	more curved and more angular distally	unique, pneumatic	(similar)	straight	?	?	unique, little marked	non-prominent: almost flat	?	?	slight undulation	?	oscine type	?	?	oscine type	very reduced
	Paradisaeidae	*Ptiloris paradiseus, Paradisaea minor*	more curved and more angular distally	unique, pneumatic	similar	straight	?	?	double, rather little marked	non-prominent: flat	?	?	extremely slight undulation	similar	oscine type	similar	?	oscine type	absent
	Other oscines		?	unique or double (22) (in Corvidae: unique, pneumatic)	similar	straight	?	?	Generally double (in Corvidae: double, well marked)	non-prominent: flat			absent or present (23) (in Corvidae: absent)		oscine type			oscine type	absent
Wieslochia weissi † (24)			?	unique	?	straight	?	?	?	?	?	?	marked	more rounded	symphysis poorly developed; presents a pointed protuberance	similar	?	?	?

Taxon				Femur	Tibiotarsus			Tarsometatarsus						Pedal phalanges	
Suborder to Family			Species (species seen for comparison are listed, but see *)	**Hollow just distal to proximal articular surface, caudal side**	**Medial crest (tbt proximalmost shaft)**	**Tiny proximo-distal groove between the tuberositas retinaculi extensorius lateralis and the tuberculum retinaculi m. fibularis (see 4), lateral side of distal end, proximal to the lateral condyle, rostral aspect (Fig. S1).**	**Caudo-distal ridge between the condyles (dist. tbt)**	**Fusion (continuity) between crista med. plantaris and crista med. hypotarsi**	**Ossified pons supratendineus**	**Position of pons supratendineus**	**Grooved inner (II) / outer (IV) trochleae tarsometatarsi (also dorsally unless stated otherwise)**	**Trochlea metatarsi II**	**Trochlea metatarsi IV**	**Notched medial phalanx (laterally)**	**Shape of claws**
Revest-des-Brousses fossil (early Oligocene) NT-LBR-014				marked	absent	Present; (NB: seems restricted distally owing to slight crushing)	hardly outlined	no ; crista plantaris relatively little developed	present (flattened)	much proximal	no	extension almost as distal as tr. mtt. III	extension almost as distal as tr. mtt. III	no	generalist
Acanthisittidae			*Acanthisitta chloris* (5)	absent	absent to present	present; short and more protruding (including laterally)	stronger	no	present	rather proximal like suboscines in general	no	markedly lesser distal extension	slightly lesser distal extension than III	no	long, narrow, more or less curved
Tyranni (Suboscines)	Eurylaimides	Pittidae	*Pitta sordida*	slighter	absent	present; less distal	slight	no; wide gap between the two crests	present	as fossil	II: only dorsal and slightly; IV: yes (plantar to partly dorsal)	as fossil	extension very slightly less distal	no	more elongated
		Philepittidae	*Philepitta castanea*	absent	slight	present; less distal	as fossil	no; crista plantaris very little developed	present	as fossil	no	as fossil	extension less distal	slightly	more elongated
		Eurylaimidae	*Cymbirhynchus macrorhynchos*	slighter	absent	rather similar	as fossil	no; wide gap between the two crests	present	as fossil	II: no; IV: yes (plantar only)	extension rather like fossil; tr. mtt. II oriented more medially	extension less distal	?	slightly more curved
		Calyptomenidae	*Calyptomena viridis*	absent	absent	present; shorter, more in relief, more distal	as fossil	no; wide gap between the two crests	present	as fossil	II: extremely slight plantaro-dist; IV: no	extension rather like fossil; tr. mtt. II oriented more medially	extension less distal	?	more curved
		Sapayoidae	*Sapayoa aenigma*	absent	absent	present; on a greater length	very slight	no; crista plantaris almost nonexistent	present	as fossil	II: no; IV: extremely slight	distal extension greater	extension slightly less distal	no	rather similar
	Tyrannides	Furnariidae (6)	*Geositta cunicularia*	marked	slight	present; less distal	slight	no; rather small gap between the two crests	present	intermediate (more distal)	II: yes; IV: no (9)	as fossil	very narrow and reduced	no	rather tapering and curved
			Dendrocincla fuliginosa	absent	slight	present; slightly less distal	slight	no; small gap between the two crests	present	as fossil	no	extension slightly less distal	extension slightly less distale; (NB: trochlea III very much incised by groove)	no	longer and more curved
		Thamnophilidae	*Myrmotherula axillaris*	shallow	almost absent	present; less restricted distally	slight	no; wide gap between the two crests	present	distal rather like in Corvidae	no	as fossil	extension less distal	no	more curved
			Sakesphorus canadensis	absent	slight	present; (less restricted distally)	slight	no; rather small gap between the two crests	present	as fossil	no	as fossil	extension as distal as tr. mtt. III	no	longer
			Thamnophilus doliatus (skull)	?	?	?	?	?	?	?	?	?	?	?	?
			Thamnophilus coecus (skull)	?	?	?	?	?	?	?	?	?	?	?	?
		Conopophagidae	*Conopophaga ardesiaca saturata*	absent	almost absent	present; less restricted distally	none	no (plantar crests grows progressively starting from hypotarsus)	present	as fossil	family: no / yes (11)	as fossil	extension slightly less distal	no	slightly narrower
		Rhinocryptidae	*Scytalopus unicolor*	marked	marked ; (and pronounced indentation proximal to the fibular crest) (11)	present; less marked and less distal	stronger (11)	no; wide gap between the two crests	present	distal rather like in Corvidae	yes (11)	slightly more distal (11)	extension less distal (11)	yes	longer, more tapering and more curved
		Formicariidae	*Formicarius analis*	very shallow	absent	present; less distal	none	no; wide gap between the two crests	present	rather proximal	yes (moreover the IV) (11)	as fossil	?	yes	tapering; little curved
		Pipridae	*Ceratopipra erythrocephala*	shallow	absent	present; less distal	slight	no; wide gap between the two crests	present	as fossil	only very slightly, only plantar	almost as distal as fossil	extension slightly less distal	no	as fossil
			Neopelma sulphureiventer	?	?	?		?	present	as fossil	?	?	?	?	?
			Tyranneutes stolzmanni	absent	absent	rather similar	very slight	no; wide gap between the two crests; crista plantaris rather little developed	present	as fossil	no	as fossil	extension as distal as tr. mtt. III	no	similar
			Chloropipo holochlora viridior	absent	very slight	present; less distal	very slight	no; wide gap between the two crests; crista plantaris rather little developed	present	as fossil	II: slightly; IV: no	as fossil	extension less distal	no	narrower, longer and more curved
			Manacus manacus	absent	absent	?	very slight	no; wide gap between the two crests; crista plantaris rather little developed	?	?	no	as fossil	extension less distal	no	slightly narrower
			Machaeropterus pyrocephalus	absent	absent	?	very slight	no; wide gap between the two crests; crista plantaris rather little developed	present	as fossil	no	as fossil	extension as distal as tr. mtt. III	no	slightly longer and more tapering
			Chiroxiphia linearis	marked	absent	present; less distal	very slight	no; wide gap between the two crests; crista plantaris rather little developed	present	as fossil	II: slightly; IV: no	as fossil	extension less distal	no	slightly longer, more curved and more tapering
			Xenopipo atronitens	marked	absent	rather similar	none	no; wide gap between the two crests; crista plantaris very little developed	present (at least in part ossified)	slightly more proximal	II: slightly (at distal tip); IV: no	as fossil	extension as distal as tr. mtt. III	no	slightly longer and more curved
			Masius chrysopterus	marked	absent	rather similar	as fossil	no; wide gap between the two crests; crista plantaris very little developed	present	as fossil	II: slightly (at distal tip); IV: no	as fossil	extension less distal	no	slightly longer, more curved and more tapering
			Antilophia galeata	absent	very slight	rather similar	as fossil	identical to fossil	present	as fossil	no	as fossil	extension less distal	no	slightly longer
		Cotingidae	*Pipreola arcuata*	?	?	?		?	?	?	?	?	?	?	?
			Cephalopterus ornatus	absent	absent	absent (or hardly visible)	slight / absent	no; wide gap between the two crests	present	as fossil	both very slightly plantar; II also visible at distal tip	as fossil	extension slightly less distal	no	slightly more curved
			Cotinga sp	absent	absent	present; less distal	slight	no; wide gap between the two crests	present	as fossil	II: no; IV: yes (essentially plantar)	almost as distal as fossil	extension less distal	?	slightly longer and more curved
			Procnias sp. (skull)	?	?	?	?	?	?	?	?	?	?	?	?
			Pipreola intermedia signata (skull)	?	?	?	?	?	?	?	?	?	?	?	?
			Rupicola rupicola	slighter	?	?	?	?	?	?	?	?	?	?	?
			Phytotoma rara	slighter	marked	rather similar		no	present	as fossil	?	?	?	no	similar
		Tityridae	*Oxyruncus cristatus*	?	?	?		?	?	?	?	?	?	?	?
			Onychorhynchus coronatus	much slighter	marked	rather similar		no	present	as fossil	no	extension less distal	extension slightly more distal	no	more curved
			Tityra semifasciata	absent	marked	rather similar		no	?	?	II: slightly; IV: no	as fossil	?	no	more curved
			Schiffornis turdina	absent	very slight	similar	very slight	no; wide gap between the two crests; crista plantaris rather little developed	present	as fossil	II: very slightly; IV: no	as fossil	extension less distal	no	longer
		Tyrannidae	*Tyrannus dominicensis*	absent	absent	present; less marked and less distal	slight	no; wide gap between the two crests	absent	-	both slightly plantar; II: also visible at distal tip	extension slightly less distal	extension less distal	?	more curved and tapering
			Tyrannus sp.	?	?	?		?			?	?	?	?	?
			Todirostrum sp.	absent	slight	rather similar		no; wide gap between the two crests; crista plantaris very little developed	absent	-	II: slightly; IV: no	almost as distal as fossil	extension less distal	?	?
			Contopus latirostris (skull)	?	?	?		?	?	?	?	?	?	?	?

		Rhynchocyclus olivaceus	absent	marked	rather similar		no; wide gap between the two crests; crista plantaris very little developed	present	as fossil	no	extension less distal	?	no	more curved and tapering
		Rhynchocyclus sp.	absent	marked	rather similar		no; wide gap between the two crests; crista plantaris very little developed	present	as fossil	no	as fossil	extension less distal	no	similar
Passeri (Oscines)	Menuridae (18)	*Menura novaehollandiae*	absent	marked; and more proximal	?	marked	no; crista plantaris little developed	present	rather proximal	yes	extension slightly more distal	extension slightly less distal	yes	?
	Atrichornithidae (19)		absent	marked	?	little to moderately marked	no; crista plantaris little developed	present	intermediate / proximal	yes	extension slightly more distal	extension slightly less distal	yes	?
	Ptilonorhynchidae (11)	*Sericulus* sp., *Ptilonorhynchus violaceus*	absent	almost absent	hardly visible	almost absent	yes	present	?	no	extension slightly less distal	extension slightly less distal	no	?
	Climacteridae (2)		?	?	?		yes (2)	?	?	?	?	?	?	?
	Meliphagidae	*Philemon corniculatus, Meliphaga lewini, Manorina melanocephala*	absent	present, moderately marked	little marked	little marked	variable: no fusion to almost fusion (apparently fusion in *Philemon*) (2)	present	?	no	slightly less distal	?	no	
	Pardalotidae	*Pardalotus punctatus*	absent	present, moderately marked	absent (or hardly visible)	very little marked	no	?	?	no	?	?	?	?
	Acanthizidae	*Gerygone flavolateralis*	?	present; marked	absent		yes	absent?	?	II: no; IV: slightly	as fossil	extension slightly less distal	?	more curved and tapering
	Pomatostomidae	*Pomatostomus temporalis trivirgatus*	absent	?	very slight	very slight	no; small gap between the two crests	present	?	?	?	?	no	?
	Paradisaeidae	*Ptiloris paradiseus, Paradisaea minor*	absent	almost absent	small	almost absent	no; wide gap between the two crests	present	?	no	oriented more medially	?	slightly	?
	Other oscines		?	absent or almost absent	Corvidae: present; less distal	absent to very slight	no (2)	generally present	Corvidae: more distal	no (or extremely slightly plantarly)	Corvidae: slightly less distal extension than III	Corvidae: very slightly less distal extension than the III	no	Corvidae: generalist
Wieslochia weissi † (24)			?	?	?		?	absent	?	?	?	?	?	slightly more robust

Supplementary Table 2 Additional characters in the fossil NT-LBR-014 and extant Tyrannida (i.e., Pipridae, Cotingidae, Tityridae and Tyrannidae).

All character states of species compared to the fossil are given relative to state of the fossil.

NB: for several modern specimens, character states are absent due to the lack of the element or element part, or the incomplete specimen preparation, obscuring detail by ligaments for instance. It is specified for which specimens the skull only could be examined.

Taxon		Skull		Humerus		Ulna	
Family	Species	**Size of nasal opening**	**Lateral parts of etecthmoid**	**Crus ventralis fossae**	**Crus dorsalis fossae**	**Cotyla dorsalis**	**Position of the dorso-proximal edge of the incisura tendinosa (for mm. extensor metacarpi ulnaris and extensor digitorum communis), distal ulna**
Revest-des-Brousses fossil (early Oligocene) NT-LBR-014		large; more than half rostrum length	gap reduced	little developed	well developed	hollow; little developed	proximal
Pipridae	*Pipra erythrocephala*	large; more than half rostrum length	gap reduced	little developed	well developed	hollow; little developed	proximal
	Neopelma sulphureiventer	large; more than half rostrum length	gap reduced	?	?	hollow; little developed	proximal
	Tyranneutes stolzmanni	large; more than half rostrum length	gap reduced	little developed	well developed	hollow; little developed	proximal
	Chloropipo holochlora viridior	large; more than half rostrum length	gap reduced	little developed	well developed	hollow; little developed	proximal
	Manacus manacus	large; more than half rostrum length	gap reduced	little developed	well developed	hollow; little developed	proximal
	Machaeropterus pyrocephalus	large; more than half rostrum length	gap reduced	little developed	well developed	hollow; little developed	proximal
	Chiroxiphia linearis	large; more than half rostrum length	gap reduced	little developed	well developed	hollow; little developed	proximal
	Xenopipo atronitens	large; more than half rostrum length	gap reduced	little developed	well developed	hollow; little developed	proximal
	Masius chrysopterus	large; more than half rostrum length	gap reduced	little developed	well developed	hollow; little developed	proximal
	Antilophia galeata	large; more than half rostrum length	gap reduced	little developed	well developed	hollow; little developed	proximal
Cotingidae	*Pipreola arcuata*	medium-sized	gap larger	well developed	well developed	flat; wide	proximal
	Cephalopterus ornatus	medium-sized	gap larger	well developed	well developed	flat; wide	proximal
	Cotinga sp.	medium-sized	gap larger	well developed	well developed	flat; wide	proximal
	Procnias sp. (skull)	medium-sized	gap larger	?	?	?	?
	Pipreola intermedia signata (skull)	medium-sized	gap larger	?	?	?	?
	Rupicola rupicola	medium-sized	gap larger	?	?	?	?
	Phytotoma rara	medium-sized	gap larger	well developed	well developed	flat; wide	proximal
Tityridae	*Oxyruncus cristatus*	small	gap larger	?	?	flat; wide	proximal
	Onychorhynchus coronatus	small	gap larger	well developed	well developed	flat; wide	proximal
	Tityra semifasciata	small	gap larger	well developed	well developed	flat; wide	proximal
	Schiffornis turdina	small	gap larger	well developed	well developed	flat; wide	proximal
Tyrannidae	*Tyrannus dominicensis*	large; more than half rostrum length	gap moderate but larger than fossil	well developed	little developed	hollow; wide	distal
	Tyrannus sp.	large; more than half rostrum length	gap moderate but larger than fossil	well developed	little developed	hollow; wide	distal
	Todirostrum sp.	large; more than half rostrum length	gap moderate but larger than fossil	well developed	little developed	hollow; wide	distal
	Contopus latirostris	large; more than half rostrum length	gap moderate but larger than fossil	?	?	hollow; wide	distal
	Rhynchocyclus olivaceus	large; more than half rostrum length	gap moderate but larger than fossil	well developed	little developed	hollow; wide	distal
	Rhynchocyclus sp.	large; more than half rostrum length	gap moderate but larger than fossil	well developed	little developed	hollow; wide	distal

Supplementary Table 3 Measurements (lengths) of the main bones of the Luberon fossil NT-LBR-014 (mm).

Skull		Wing		Leg	
Cranium	21.5	Humerus	19.4	Femur	18.6
Beak	15.1	Ulna	25.2	Tibiotarsus	28.7
		Carpometacarpus	13.6	Tarsometatarsus	20.4
Scapular belt		Wing Phalanx 1 of major digit	6.5	Digit III	16.8
Coracoid	15.7	Total length of wing bones	64.7	Total length of leg bones	84.5
Scapula	19.6				

Supplementary Table 4 States of characters selected and coded for the radial visualisation in Fig. 5. The character states are coded in order to categorize observations in Supplementary Tables 1 and 2, as follows. State (0) corresponds to absence of the character, state (1) to presence of the character but state different from the fossil's, and state (2) to presence of character and state similar or identical to the fossil's. The extant species of comparison are limited here to the Tyrannides for which all characters were assessable, and the characters are those exhibiting heterogeneous states across the Tyrannides.

	Skull							Coracoid				Scapula	Humerus				Ulna	Carpometacarpus					Proximal wing phalanx of major digit		Femur	Tibiotarsus	Tarsometatarsus			
	Cranium shape (lateral profile)	Feather crest	Relative size of orbits	Osseous nasal septum	Fenestra antorbitalis (proportion)	latero-dorsal part of ectethmoid	Rostrum and mandible (mdb)	Processus procoracorideus	Processus acrocoracoideus	Brachial tuberosity (tuberculum brachiale)	Coracoid blade shape	Outline	Crista deltopectoralis	Shaft shape	Depth of incisura capitis	Processus supracondylaris dorsalis	Papillae remigalis caudalis	Processus dentiformis	Processus Intermetacarpalis	Distal symphysis of os metacarpale minus	carpometacarpus outline	Processus extensorius	Blade	Processus internus indicis	Hollow just distal to proximal articular surface, caudal side	Incisura intercondylaris	Proximal tarsometatarsus	Position of pons supratendineusineus	Trochlea metatarsus II	Trochlea metatarsus IV
**Fossil**	2	2	2	2	2	2	2	2	2	2	2	2	2	2	2	2	2	2	2	2	2	2	2	2	2	2	2	2	2	2
Geositta cunicularia	2	1	1	0	1	1	1	1	1	1	1	1	2	2	1	1	1	0	1	1	1	2	1	2	2	1	1	1	2	2
Dendrocincla filignosa	1	0	1	0	1	2	1	1	1	1	1	1	2	2	1	1	1	0	1	1	1	2	1	0	0	1	1	2	1	2
Myrmotherula axillaris	2	1	1	1	1	1	1	1	1	1	1	1	2	2	1	1	2	1	1	1	1	2	1	0	0	1	1	1	2	2
Sakesphorus canadensis	1	1	1	1	1	1	1	1	1	1	1	1	2	2	1	1	2	0	1	1	1	2	1	0	0	1	1	2	2	1
Conopophagidae ardesiaca	1	0	1	1	1	1	1	1	1	1	1	1	2	2		1	2	1	1	1	1	2	1	0	0	1	1	2	2	2
Scytalopus unicolor	1	1	1	0	1	1	1	1	1	1	1	1	1	1	1	2	1	1	1	1	1	2	1	1	2	1	1	1	2	2
Formicarius analis	1	0	1	0	1	1	1	1	1	1	1	1	2	1	1	1	1	1	1	1	1	1	1	1	0	1	1	1	2	2
Pipra erythrocephala	2	1	1	1	1	1	2	1	2	1	1	1	2	2	1	2	1	0	1	2	1	2	1	2	1	1	1	2	2	2
Tyranneutes stolzmanni	2	1	2	1	1	1	1	1	1	1	2	1	2	2	1	2	1	0	1	1	1	2	1	2	0	2	2	2	2	1
Chloropipo holochlora	1	1	1	1	1	1	1	2	1	1	1	1	2	2	1	2	1	0	1	1	1	2	2	2	0	1	2	2	2	2
Manacus manacus	1	1	1	1	1	1	1	1	1	1	1	1	2	2	1	2	1	2	1	2	1	2	2	0	0	2	2	2	2	2
Machaeropterus pyrocephalus	1	1	1	1	1	1	1	1	1	1	2	2	2	2	1	2	1	1	1	2	1	2	1	2	0	2	2	2	2	1
Chiroxiphia linearis	1	1	1	1	1	1	1	1	1	1	2	2	2	2	1	2	2	0	2	2	2	2	1	2	2	1	2	2	2	2
Xenopipo atronitens	1	1	1	1	2	2	1	2	1	1	1	1	2	2	1	2	1	1	2	1	2	2	2	2	2	2	2	2	2	1
Masius chrysopterus	1	1	1	1	1	2	1	1	1	1	2	1	2	2	1	2	1	1	1	2	1	2	2	1	2	2	2	2	2	2
Antilophia galeata	2	2	1	1	1	1	2	2	2	2	2	2	2	2	1	2	2	0	2	2	2	2	2	2	0	2	2	2	2	2
Cephalopterus ornatus	1	1	1	1	1	1	1	1	2	2	1	1	2	2	1	1	1	1	1	1	1	2	1	2	0	0	1	2	2	2
Cotinga sp	1	1	1	1	1	2	1	1	1	2	2	1	2	2	1	1	1	0	2	1	2	1	2	2	0	1	1	2	1	2
Onychorynchus coronatus	1	1	1	1	1	1	1	1	1	1	1	1	2	2	1	1	2	1	1	1	1	2	1	2	1	2	1	2	1	1
Schiffornis turdina	2	1	1	1	1	1	1	1	1	1	1	1	2	2	1	2	1	1	1	1	1	2	2	2	0	2	2	2	2	2
Tyrannus dominicensis	2	1	1	1	1	1	1	2	1	1	1	2	2	2	1	1	1	1	1	1	1	2	2	2	0	1	1	0	1	2
Todirostrum sp.	2	0	1	1	1	1	1	1	1	1	1	1	2	2	1	1	2	0	2	1	2	2	2	2	0	1	1	0	1	2
Rhynchocyclus olivaceus	2	0	1	1	1	1	1	1	1	2	1	1	2	2	1	1	1	1	1	1	1	2	1	2	0	2	1	2	1	1
Rhynchocyclus sp.	2	1	1	1	1	1	1	1	1	1	1	1	2	2	1	0	1	1	1	1	2	2	1	2	0	2	1	2	1	1

Supplementary Table 5 Character matrix used for the phylogenetic analyses.

	Feather crest	Relative size of orbits	Shape of orbits	Size of nasal opening	Fenestra antorbitalis (proportion)	Os lacrimale	Latero-dorsal part of ectethmoid	Lateral parts of etecthmoid	Crest/ridge on culmen	Foramen (foramina) of coracoid omal end	Processus procoracoideus	Processus acrocoracoideus	Brachial tuberosity (tuberculum brachiale)	Sulcus medialis supracoracoidei	Fossa pneumotricipitalis	Crista deltopectoralis	Crus ventralis fossae	Crus dorsalis fossae	Shaft shape	Depth of incisura capitis	Processus flexorius	Processus supracondylaris dorsalis
Fossil	2	0	2	0	1	2	3	0	0	0	1	1	2	2	1	0	0	1	1	2	1	1
Pitta sordida	0	0	0	?	1	0	1	?	0	0	1	1	0	0	1	0	?	?	1	1	0	1
Philepitta castanea	0	0	0	?	1	0	0	?	0	0	1	0	?	?	1	0	?	?	1	1	?	1
Cymbirhynchus macrorhynchos	0	0	0	?	0	0	2	?	0	0	0	0	0	0	1	0	?	?	1	1	0	1
Calyptomena viridis	1	0	0	?	0	0	2	?	1	0	1	0	0	0	1	0	?	?	1	1	0	1
Sapayoa aenigma	0	0	0	?	1	0	0	?	0	0	1	1	?	?	1	0	?	?	1	0	0	1
Geositta cunicularia	0	0	0	?	0	1	2	?	0	0	0	1	0	2	1	0	?	?	1	0	1	1
Myrmotherula axillaris	0	1	1	?	0	1	1	?	0	?	0	1	1	1	1	0	?	?	1	?	?	1
Sakesphorus canadensis loretoyacuensis	0	0	1	?	0	1	2	?	0	?	0	1	?	?	1	0	?	?	1	?	?	1
Conopophaga ardesiaca saturata	0	0	1	?	0	1	0	?	0	?	0	1	0	2	1	0	?	?	1	?	?	1
Scytalopus unicolor	0	1	0	?	0	1	1	?	1	1	0	0	0	2	1	1	?	?	0	1	1	1
Formicarius analis	0	0	1	?	0	1	1	?	0	0	0	0	0	1	1	0	?	?	1	1	1	1
Dendrocincla fuliginosus	0	0	1	?	0	1	3	?	0	0	1	1	1	1	1	0	?	?	1	1	?	1
Pipra erythrocephala	1	0	2	0	0	2	1	0	0	0	1	1	?	?	1	0	0	1	1	1	1	1
Neopelma sulphureiventer	0	1	1	0	0	?	1	0	0	0	1	1	1	1	?	?	0	1	?	?	?	?
Tyranneutes stolzmanni	0	0	2	0	0	?	1	0	0	0	1	1	1	2	1	0	0	1	1	1	1	1
Chloropipo holochlora viridior	0	0	1	0	1	?	1	0	0	0	1	1	1	1	1	0	0	1	1	0	1	1
Manacus manacus trinitatis	0	0	1	0	0	?	2	0	0	?	1	1	1	1	1	0	0	1	1	0	1	1
Machaeropterus pyrocephalus pyrocephalus	0	0	1	0	0	?	1	0	0	?	1	1	0	2	1	0	0	1	1	1	1	1
Chiroxiphia linearis	0	0	1	0	0	2	1	0	0	?	1	1	1	1	1	0	0	1	1	1	1	1
Xenopipo atronitens	0	0	1	0	1	2	3	0	0	0	1	1	1	2	1	0	0	1	1	0	1	1
Masius chrysopterus	1	0	1	0	0	2	3	0	0	0	1	0	1	2	1	0	0	1	1	0	1	1
Antilophia galeata	2	0	1	0	0	2	1	0	0	0	1	1	2	2	1	0	0	1	1	1	1	1
Pipreola arcuata	0	0	1	1	0	2	1	2	0	0	1	1	1	1	1	0	1	1	1	1	1	1
Cephalopterus ornatus	1	0	1	1	0	2	1	2	0	0	0	1	2	1	1	0	1	1	1	0	1	1
Cotinga sp	0	0	0	1	1	2	2	2	0	0	0	1	2	0	1	0	1	1	1	0	1	1
Phytotoma rara	0	0	1	1	0	2	1	2	0	0	0	1	1	1	1	0	1	1	1	1	1	1
Onychorynchus coronatus	1	0	1	2	0	2	1	2	0	0	0	0	1	1	1	0	1	1	1	1	1	1
Tityra semifasciata	0	0	1	2	0	0	1	2	0	0	0	0	1	0	1	0	1	1	1	1	1	1
Schiffornis turdininus amazonus	0	0	1	2	0	2	1	2	0	0	1	0	1	1	1	0	1	1	1	1	1	1
Tyrannus dominicensis	0	0	?	0	?	2	1	1	0	0	1	0	0	0	1	0	1	0	1	1	1	1
Todirostrum sp	0	0	1	0	0	0	0	1	0	0	0	0	1	1	1	0	1	0	1	0	1	1
Rhynchocyclus olivaceus	0	0	1	0	0	0	1	1	0	0	0	0	2	1	1	0	1	0	1	0	1	1
Rhynchocyclus sp	1	0	1	0	0	0	1	1	0	0	1	0	1	1	1	0	1	0	1	0	1	1
Acanthisitta chloris	0	1	1	?	1	0	0	?	0	1	0	0	?	?	0	0	?	?	0	0	?	1
Gerygone flavolateralis	0	0	0	?	0	1	0	?	0	1	0	0	2	1	0	0	?	?	1	1	1	0

	Processus supracondylaris dorsalis	Tuberculum ligamenti collateralis ventralis	Cotyla ventralis	Cotyla dorsalis	Position of the dorso-proximal edge of the incisura tendinosa (for mm extensor metacarpi ulnaris and extensor digitorum communis), distal ulna	Papillae remigales caudales	Processus dentiformis	Position of processus intermetacarpalis	Distal symphysis of os metacarpale minus	Processus extensorius	Blade	Processus internus indicis	Hollow just distal to proximal articular surface, caudal side	Medial crest (tibiotarsis proximal most shaft)	Tiny proximo-distal groove between the tuberositas retinaculi extensorius lateralis and the tuberculum retinaculi m fibularis, lateral side of distal end, proximal to the lateral condyle, rostral aspect	Fusion (continuity) between crista med plantaris and crista med hypotarsi	Ossified pons supratendineus	Position of pons supratendineus	Trochlea metatarsi II	Trochlea metatarsi IV
Fossil	2	1	1	0	0	1	2	0	1	0	0	0	2	0	2	0	1	2	0	1
Pitta sordida	2	1	0	?	?	3	2	0	1	1	1	0	1	0	1	0	1	2	0	2
Philepitta castanea	2	1	?	?	?	3	1	0	1	1	1	0	0	1	1	0	1	2	0	?
Cymbirhynchus macrorhynchos	1	1	0	?	?	3	2	0	1	1	0	0	1	0	1	0	1	2	1	2
Calyptomena viridis	1	1	0	?	?	3	1	0	1	1	0	0	0	0	1	0	1	2	1	2
Sapayoa aenigma	2	1	0	?	?	2	2	0	1	1	0	0	0	0	1	0	1	2	2	?
Geositta cunicularia	1	1	?	?	?	0	1	0	1	0	1	0	2	1	1	0	1	4	0	?
Myrmotherula axillaris	1	1	?	?	?	1	1	0	1	?	2	1	1	0	1	0	1	3	0	1
Sakesphorus canadensis loretoyacuensis	1	1	?	?	?	1	0	0	1	?	1	1	0	1	1	0	1	2	0	0
Conopophaga ardesiaca saturata	2	1	?	?	?	1	3	0	1	0	1	1	0	0	1	0	1	2	0	1
Scytalopus unicolor	2	1	?	?	?	0	3	0	1	0	1	1	2	0	1	0	1	3	0	1
Formicarius analis	2	1	?	?	?	2	2	0	1	2	1	1	0	0	1	0	1	1	0	?
Dendrocincla fuliginosus	1	1	?	?	?	1	1	0	1	0	1	1	0	1	1	0	1	2	1	1
Pipra erythrocephala	2	1	1	0	0	0	0	0	1	0	0	0	1	0	1	0	1	2	0	1
Neopelma sulphureiventer	?	1	1	0	0	0	0	0	1	0	?	?	?	?	?	?	?	?	?	?
Tyranneutes stolzmanni	2	1	?	0	0	0	0	0	1	0	0	0	0	0	2	0	1	2	0	0
Chloropipo holochlora viridior	2	1	?	0	0	1	0	0	1	0	0	0	0	0	1	0	1	2	0	1
Manacus manacus trinitatis	2	1	?	0	0	1	2	0	1	0	0	1	0	0	?	0	?	?	0	1
Machaeropterus pyrocephalus pyrocephalus	2	1	?	0	0	3	1	0	1	0	0	0	0	0	?	0	1	2	0	0
Chiroxiphia linearis	2	1	?	0	0	1	0	0	1	0	0	0	2	0	1	0	1	2	0	1
Xenopipo atronitens	2	1	?	0	0	0	2	0	1	0	0	0	2	0	2	0	1	1	0	0
Masius chrysopterus	2	1	1	0	0	0	1	0	1	0	0	0	2	0	2	0	1	2	0	1
Antilophia galeata	2	1	1	0	0	1	0	0	1	0	0	0	1	0	2	0	1	2	0	?
Pipreola arcuata	2	1	1	1	0	?	1	0	1	0	0	0	?	?	?	?	?	?	?	?
Cephalopterus ornatus	1	1	1	1	0	3	0	0	1	0	0	0	0	0	0	0	1	2	0	1
Cotinga sp	1	1	1	1	0	2	0	0	1	2	0	0	0	0	1	0	1	2	1	1
Phytotoma rara	1	1	1	1	0	0	1	0	1	?	0	0	1	1	2	0	1	2	?	?
Onychorynchus coronatus	1	1	1	1	0	1	1	0	1	0	0	0	1	1	2	0	1	2	1	2
Tityra semifasciata	2	1	1	1	0	0	0	0	1	0	0	0	0	1	2	0	?	?	0	?
Schiffornis turdininus amazonus	2	1	?	1	0	0	1	0	1	0	0	0	0	0	2	0	1	2	0	1
Tyrannus dominicensis	1	1	1	1	1	2	1	0	1	0	0	0	0	0	0	0	0	0	1	1
Todirostrum sp	1	1	1	1	1	1	0	0	1	0	0	0	0	1	2	0	0	0	1	1
Rhynchocyclus olivaceus	1	1	1	1	1	0	1	0	1	0	0	0	0	1	2	0	1	2	1	?
Rhynchocyclus sp	0	1	1	1	1	0	1	0	1	0	0	0	0	1	2	0	1	2	1	2
Acanthisitta chloris	0	1	?	?	?	2	0	1	1	?	2	1	0	1	1	0	1	1	1	?
Gerygone flavolateralis	2	0	0	?	?	2	3	1	0	0	?	?	?	1	0	1	0	0	1	0

References (for Supplementary Table 1)

1. del Hoyo, J., Elliott, A., Sargatal, J., Christie, D. A. & de Juana, E. (eds) *Handbook of the Birds of the World Alive* (Lynx Edicions, Barcelona, 2019). (retrieved from https://www.hbw.com/ on 13 March 2019).

2. James, H. F., Ericson, P. G. P., Slikas, B., Lei, F. M., Gill, F. B. & Olson, S. L. *Pseudopodoces humilis*, a misclassified terrestrial tit (Paridae) of the Tibetan Plateau: evolutionary consequences of shifting adaptive zones. *Ibis* **145**, 185–202 (2003).

3. Mourer-Chauviré, C., Hugueney, M. & Jonet, P. Découverte de Passeriformes dans l'Oligocène supérieur de France. *C. R. Acad. Sci. Paris, Série II* **309**, 843–849 (1989).

4. Boles, W. E. A new songbird (Aves: Passeriformes) from the mid-Cenozoic of Riversleigh, northwestern Queensland. *Alcheringa* Special Issue **1**, 31–37 (2006).

5. Millener, P. R. Contributions to New Zealand's Late Quaternary avifauna. 1: *Pachyplichas*, a new genus of wren (Aves: Acanthisittidae), with two new species. *Journal of the Royal Society of New Zealand* **18**, 383–406 (1988).

6. Millener, P. R. & Worthy, T. H. Contributions to New Zealand's Late Quaternary avifauna. 2: *Dendroscansor decurvirostris*, a new genus and species of wren (Aves: Acanthisittidae). *Journal of the Royal Society of New Zealand* **21**, 179–200 (1991).

7. Fjeldsa, J., Irestedt, M. & Ericson, P. G. P. Molecular data reveal some major adaptational shifts in the early evolution of the most diverse avian family, the Furnariidae. *J. Ornithol.* **146**, 1–13 (2005).

8. Tonni, E. P. & Noriega, J. I. Una especie extinta de *Pseudoseisura* Reichenbach 1853 (Passeriformes: Furnariidae) del Pleistoceno de la Argentina: comentarios filogeneticos. *Ornitologia Neotropical* **12**, 29–44 (2001).

9. Feduccia, A. & Olson, S. L. Morphological similarities between the Menurae and the Rhinocryptidae, relict passerine birds of the Southern Hemisphere. *Smithsonian Contributions to Zoology* **366**, 1–22 (1982).

10. Rich, P. V., McEvey, A. R. & Baird, R. F. Osteological comparison of the scrub-birds, *Atrichornis*, and Lyrebirds, *Menura* (Passeriformes: Atrichornithidae and Menuridae). *Records of the Australian Museum* **37**, 165–191 (1985).

11. Bock, W. J. Relationships between the birds of paradise and the bower birds. *The Condor* **65**, 91–125 (1963).

12. Olson, S. L., Parkes, K. C., Clench, M. H. & Borecky, S. R. The affinities of the New Zealand passerine genus *Turnagra*. *Notornis* **30**, 319–336 (1983).

13. Zuccon, D. & Ericson, P. G. P. Molecular and morphological evidences place the extinct New Zealand endemic *Turnagra capensis* in the Oriolidae. *Mol. Phylogen. Evol.* **62**, 414–426 (2012).

14. Bock, W. J. The pneumatic fossa of the humerus in the Passeres. *The Auk* **79**, 425–443 (1962).

15. Pocock, T. N. Osteology. Contributions to the osteology of African birds. *Proceedings of the Second Pan African Ornithological Congress*, 83–94 (1966).

16. Harrison, C. J. O. Additional information on the carpometacarpal process as a taxonomic character. *Bulletin B.O.C.* **89**, 27–29 (1969).

17. Mayr, G. & Manegold, A. The oldest european fossil songbird from the Early Oligocene of Germany. *Naturwissenschaften* **91**, 173–177 (2004).

18. Mayr, G. & Manegold, A. New specimens of the earliest European passeriform bird. *Acta Palaeontologica Polonica* **51**, 315–323 (2006).

```
#NEXUS
BEGIN TAXA;
        DIMENSIONS NTAX=36;
        taxlabels


Fossil
Pitta_sordida
Philepitta_castanea
Cymbirhynchus_macrorhynchos
Calyptomena_viridis
Sapayoa_aenigma
Geositta_cunicularia
Myrmotherula_axillaris
Sakesphorus_canadensis_loretoyacuensis
Conopophaga_ardesiaca_saturata
Scytalopus_unicolor
Formicarius_analis
Dendrocincla_fuliginosa
Pipra_erythrocephala
Neopelma_sulphureiventer
Tyranneutes_stolzmanni
Chloropipo_holochlora_viridior
Manacus_manacus_trinitatis
Machaeropterus_pyrocephalus_pyrocephalus
Chiroxiphia_linearis
Xenopipo_atronitens
Masius_chrysopterus
Antilophia_galeata
Pipreola_arcuata
Cephalopterus_ornatus
Cotinga_sp
Phytotoma_rara
Onychorynchus_coronatus
Tityra_semifasciata
Schiffornis_turdina_amazonum
Tyrannus_dominicensis
Todirostrum_sp
Rhynchocyclus_olivaceus
Rhynchocyclus_sp
Acanthisitta_chloris
Gerygone_flavolateralis
;
end;

BEGIN CHARACTERS;
        DIMENSIONS NCHAR=42;
        FORMAT SYMBOLS = "0 1 2 3 4" GAP=- MISSING = ?;
        Charlabels
Feathercrest
Sizeorbits
Formorbits
Taillenarine
```

```
Fenestraantorb
Lacrimal
LatDorsEctethmoid
LatEtectmoid
Crestculmen
Foramencor
Procoracorideus
Acrocoracoideus
Tubbrachiale
Sulsupracoracoidei
Fossapneum
Cristadeltopect
Crusventral
Crusdorsal
Curvedhum
Profcapitis
Procflexorius
Procsupcondyldors
Procsupcondyldors
Ulna
Cotylavent
Cotyladors
Tubconddors
Papillae
Procdent
Positionprocintcarp
Osmetacminus
Procextens
Lameborder
Procindicis
Fossacaud
Medcrest
Incintcondy
Hypotarsi
Ponttmt
Positionponttmt
Trochlint
Trochlext
;
        MATRIX

Fossile 3       1       3       1       2       3       4
1       1       1       2       2       3       3       2
1       1       2       2       3       2       2       3
2       2       1       1       2       3       1       2
1       1       1       3       1       3       1       2
2       1       2
Pitta_sordida   1       1       1       ?       2       1
2       ?       1       1       2       2       1       1
2       1       ?       ?       2       2       1       2
3       2       1       ?       ?       4       3       1
2       2       2       1       2       1       2       1
2       2       1       3
Philepitta_castanea     1       1       1       ?       2
```

1 1 ? 1 1 2
1 ? ? 2 1 ? ? 2
2 ? 2 3 2 ? ? ?
4 2 1 2 2 2 1 1
2 2 1 2 2 1 ?

Cymbirhynchus_macrorhynchos 1 1 1 ?
1 1 3 ? 1 1 1 1
1 1 2 1 ? ? 2 2
1 2 2 2 1 ? ? 4
3 1 2 2 1 1 2 1
2 1 2 2 2 3
Calyptomena_viridis 2 1 1 ? 1
1 3 ? 2 1 2 1 1
1 2 1 ? ? 2 2 1
2 2 2 1 ? ? 4 2
1 2 2 1 1 1 1 2
1 2 2 2 3
Sapayoa_aenigma 1 1 1 ? 2 1
1 ? 1 1 2 2 ? ?
2 1 ? ? 2 1 1 2
3 2 1 ? ? 3 3 1
2 2 1 1 1 1 2 1
2 2 3 ?
Geositta_cunicularia 1 1 1 ? 1
2 3 ? 1 1 1 2 1
3 2 1 ? ? 2 1 2
2 2 2 ? ? ? 1 2
1 2 1 2 1 3 2 2
1 3 4 1 ?
Myrmotherula_axillaris 1 2 2 ? 1
2 2 ? 1 ? 1 2 2
2 2 1 ? ? 2 ? ?
2 2 2 ? ? ? 2 2
1 2 ? 3 2 2 1 2
1 4 3 1 2
Sakesphorus_canadensis_loretoyacuensis 1 1
2 ? 1 2 3 ? 1 ?
1 2 ? ? 2 1 ? ?
2 ? ? 2 2
2 ? ? ? 2 1 1
2 ? 2 2 1 2 2 1
2 2 1 1
Conopophaga_ardesiaca_saturata 1 1 2 ?
1 2 1 ? 1 ? 1 2
1 3 2 1 ? ?
2 ? ? 2 3
2 ? ? ? 2 4 1 2
1 2 2 1 1 2 1 2
2 1 2
Scytalopus_unicolor 1 2 1 ? 1
2 2 ? 2 2 1 1 1
3 2 2 ? ? 1 2 2
2 3 2 ? ? ? 1 4

```
1	2	1	2	2	3	1	2
1	4	3	1	2
Formicarius_analis	1	1	2	?	1
2	2	?	1	1	1	1	1
2	2	1	?	?	2	2	2
2	3	2	?	?	?	3	3
1	2	3	2	2	1	1	2
1	3	1	1	?
Dendrocincla_fuliginosus	1	1	2	?	1
2	4	?	1	1	2	2	2
2	2	1	?	?	2	2	?
2	2	2	?	?	?	2	2
1	2	1	2	2	1	2	2
1	2	2	2	2
Pipra_erythrocephala	2	1	3	1	1
3	2	1	1	1	2
2	?	?	2	1	1	2	2
2	2	2	3	2	2	1	1
1	1	1	2	1	1	1	2
1	2	1	2	2	1	2

Neopelma_sulphureiventer	1	2	2	1
1	?	2	1	1	1	2	2
2	2	?	?	1
2	?	?	?	?	?	2	2
1	1	1	1	1	2
1	?	?	?	?	?	?	?
?	?	?
Tyranneutes_stolzmanni	1	1	3	1
1	?	2	1	1	1	2	2
2	3	2	1	1	2	2	2
2	2	3	2	?	1	1	1
1	1	2	1	1	1	1	1
3	1	2	2	1	1
Chloropipo_holochlora_viridior	1	1	2	1
2	?	2	1	1	1	2	2
2	2	2	1	1	2	2	1
2	2	3	2	?	1	1	2
1	1	2	1	1	1	1	1
2	1	2	2	1	2
Manacus_manacus_trinitatis	1	1	2	1
1	?	3	1	1	?	2	2
2	2	2	1	1	2	2	1
2	2	3	2	?	1	1	2
3	1	2	1	1	2	1
1	?	1	?	?	1	2

Machaeropterus_pyrocephalus_pyrocephalus	1	1	2
1	1	?	2	1	1	?	2
2	1	3	2	1	1	2	2
2	2	2	3	2	?	1	1
4	2	1	2	1	1	1	1
1	?	1	2	2	1	1
```

Chiroxiphia_linearis 1 1 2 1 1
3 2 1 1 ? 2 2 2
2 2 1 1 2 2 2 2
2 3 2 ? 1 1 2 1
1 2 1 1 1 3 1 2
1 2 2 1 2
Xenopipo_atronitens 1 1 2 1 2
3 4 1 1 1 2 2 2
3 2 1 1 2 2 1 2
2 3 2 ? 1 1 1 3
1 2 1 1 1 3 1 3
1 2 1 1 1
Masius_chrysopterus 2 1 2 1 1
3 4 1 1 1 2 1 2
3 2 1 1 2 2 1 2
2 3 2 2 1 1 1 2
1 2 1 1 1 3 1 3
1 2 2 1 2
Antilophia_galeata 3 1 2 1 1
3 2 1 1 1 2 2 3
3 2 1 1 2 2 2 2
2 3 2 2 1 1 2 1
1 2 1 1 1 2 1 3
1 2 2 1 ?
Pipreola_arcuata 1 1 2 2 1 3
2 3 1 1 2 2 2 2
2 1 2 2 2 2 2 2
3 2 2 2 1 ? 2 1
2 1 1
1 ? ? ? ? ? ? ?
?
Cephalopterus_ornatus 2 1 2 2 1
3 2 3 1 1 1 2 3
2 2 1 2 2 2 1 2
2 2 2 2 2 1 4 1
1 2 1 1 1 1 1 1
1 2 2 1 2
Cotinga_sp 1 1 1 2 2 3
3 3 1 1 1 2 3 1
2 1 2 2 2 1 2 2
2 2 2 2 1 3 1 1
2 3 1 1 1 1 2 1
2 2 2 2
Phytotoma_rara 1 1 2 2 1 3
2 3 1 1 1 2 2 2
2 1 2 2 2 2 2 2
2 2 2 2 1 1 2 1
2 ? 1 1 2 2 3 1
2 2 ? ?
Onychorynchus_coronatus 2 1 2 3 1
3 2 3 1 1 1 1 2
2 2 1 2 2 2 2 2
2 2 2 2 2 1 2 2
1 2 1 1 1 2 2 3

```
1	2	2	2	3
Tityra_semifasciata	1	1	2	3	1
1	2	3	1	1	1	1	2
1	2	1	2	2	2	2	2
2	3	2	2	2	1	1	1
1	2	1	1	1	1	2	3
1	?	?	1	?
Schiffornis_turdininus_amazonus	1	1	2	3
1	3	2	3	1	1	2	1
2	2	2	1	2	2	2	2
2	2	3	2	?	2	1	1
2	1	2	1	1	1	1	1
3	1	2	2	1	2
Tyrannus_dominicensis	1	1	?	1	?
3	2	2	1	1	2	1	1
1	2	1	2	1	2	2	2
2	2	2	2	2	2	3	2
1	2	1	1	1	1	1	1
1	1	0	2	2
Todirostrum_sp	1	1	2	1	1	1
1	2	1	1	1	1	2	2
2	1	2	1	2	1	2	2
2	2	2	2	2	2	1	1
2	1	1	1	1	2	3	1
1	0	2	2
Rhynchocyclus_olivaceus	1	1	2	1	1
1	2	2	1	1	1	1	3
2	2	1	2	1	2	1	2
2	2	2	2	2	2	1	2
1	2	1	1	1	1	2	3
1	2	2	2	?
Rhynchocyclus_sp	2	1	2	1	1	1
2	2	1	1	2	1	2	2
2	1	2	1	2	1	2	2
1	2	2	2	2	1	2	1
2	1	1	1	1	2	3	1
2	2	2	3
Acanthisitta_chloris	1	2	2	?	2
1	1	?	1	2	1
1	?	?	1	1	?	?	1
1	?	2	1	2	?	?	?
3	1	2	2	?	3	2	1
2	2	1	2	1	2	?

Gerygone_flavolateralis	1	1	1	?	1
2	1	?	1	2	1	1	3
2	1	1	?	?	2	2	2
1	3	1	1	?	?	3	4
2	1	1	?	?	?	2	1
2	1	0	2	1	;
END;

log/file=resultats-2.txt;
Begin paup;
```

```
        outgroup Acanthisitta_chloris Gerygone_flavolateralis;
        hsearch swap=tbr addseq=random;
        roottrees/root=outgroup;
        savetrees/file=tree2.tre;
        describetrees/fvalue apolist;
end;
```
